# Quantifying mean, variability, and uncertainty in indoor radon exposure in Pennsylvania using random forest and quantile regression forest models

**DOI:** 10.1038/s41598-026-37891-3

**Published:** 2026-03-05

**Authors:** Heechan Lee, Dakotah Maguire, Jeremy Logan, Greeshma Agasthya, Shaheen Dewji, Heidi A. Hanson

**Affiliations:** 1 Nuclear and Radiological Engineering and Medical Physics Programs, George W. Woodruff School of Mechanical Engineering, Georgia Institute of Technology, 770 State Street, Atlanta, GA 30332 USA; 2 Advanced Computing for Health Sciences Section, Oak Ridge National Laboratory, 1 Bethel Valley Road, Oak Ridge, TN 37830 USA; 3 Department of Environmental and Occupational Health, University of California, Irvine, 856 Health Sciences Quad, Irvine, GA 92697 USA; 4 Data Engineering Group, Data and AI Section, Oak Ridge National Laboratory, 1 Bethel Valley Road, Oak Ridge, TN 37830 USA

**Keywords:** Radon, Geology, Prediction model, Machine learning, ZCTA-level predictions, Environmental health, Exposome, Environmental sciences, Risk factors

## Abstract

Radon is a naturally occurring radioactive gas that poses a serious health risk as the primary cause of lung cancer in non-smokers. Despite the well-known adverse association with health outcomes, current radon exposure assessments are limited to county-level or average-level estimates, which fail to capture regional variability. This study uses Machine Learning models, including Random Forest (RF) and Quantile Regression Forest (QRF), to estimate the indoor radon concentrations at the ZCTA (Zip code tabulation area)-level and characterize uncertainties in model estimates. Incorporating geological, meteorological, and building-specific data, the models aim to improve radon risk assessment by capturing mean exposure, variability, and extreme concentration levels. Processed radon test data (*n* = 718,111) were analyzed using average, variability, and quantile prediction methods. Models that estimate the average radon exposure at the ZCTA-level can yield promising model-fit results, but they do not capture the underlying variability of indoor radon exposure within a ZCTA. We utilize volatility analyses to identify characteristics indicative of high variability of indoor radon exposure. We also show that a QRF model can be used to estimate upper quantiles of residential radon exposure, thereby uncovering localized areas of elevated exposure that were not apparent in mean estimates. The results highlighted the need for a deep characterization of exposure risk and show that regions with moderate average exposure levels could still harbor extreme outliers with implications for evaluating health risks. Utilizing multiple radon exposure models allows for a deeper characterization of radon risk within a geographic area and can better identify high-risk areas. The results from this study provide a foundation for developing mitigation strategies and examining associations between radon exposure and health outcomes at fine scales. Future research should extend the geographic scope and incorporate additional environmental risk factors to establish a comprehensive framework for risk assessment.

**Supplementary Information:**

The online version contains supplementary material available at 10.1038/s41598-026-37891-3.

## Introduction

As a naturally occurring radioactive gas, radon is a major contributor to background radiation exposure^1^ and a significant health concern as the second leading cause of lung cancer overall and the leading cause of lung cancer in nonsmokers^2^. Seeping undetected into dwellings through the ground, this colorless and odorless gas poses unique risks because radon’s decay products can attach to lung tissue and emit ionizing radiation that initiates carcinogenic processes^3^. Recent studies have also suggested potential links between radon exposure and cardiovascular disease^4,5^ and its potential interaction with PM 2.5,^4,6^ findings that could broaden the understanding of radon’s health impacts.

Despite the well-established links between radon exposure and adverse health outcomes,^7–10^ radon measurement and exposure assessment often involve significant uncertainties^11,12^. Indoor radon concentrations can vary significantly, even between neighboring houses, owing to differences in soil composition, building materials, and ventilation systems^13,14^. Moreover, radon estimates are currently limited to county-level data, which insufficiently captures the localized variability of radon exposure. This limitation poses challenges for effective public health interventions and epidemiological research.

This study applied multiple machine learning models to estimate indoor radon concentrations at the zip-code scale and examined factors contributing to uncertainty in these estimates. This approach hypothesizes that integrating geological, outdoor meteorological, and building-specific factors improves the accuracy of radon concentration predictions at the zip-code scale while identifying areas where mean or median predictions may underestimate exposure for a large percentage of the population. By combining these models, this study offers a more refined approach for radon risk assessment and targeted mitigation strategies. Estimating indoor radon exposure at finer spatial scales and quantifying the uncertainty in these estimates can also enhance the precision of environmental exposure data for epidemiological studies investigating the links between indoor radon exposure and health outcomes. Indoor radon tests were used to estimate potential for indoor radon exposure at the zip-code tabulation area (ZCTA) scale, with the overarching goal of providing more accurate assessments of risks posed by indoor radon exposure.

## Background

### Health effects of radon

Radon is a radioactive gas formed from the natural decay of uranium, which is commonly found in rocks, soil, and certain building materials. Inhalation of radon and its progeny exposes biological tissue to ionizing radiation, which has the potential to induce DNA damage and increase the risk of cancer, with lung cancer being the highest potential risk^8,15^. Radon-222, the isotope predominantly linked with radon-induced health risks, has a half-life of ~ 3.8 days and produces radioactive progeny, including polonium-218 and polonium-214. Because radon gas is colorless and odorless, specialized instruments are required to detect and measure it.

### Sources of indoor radon

The primary sources of indoor radon are geologic materials and building materials. Radon is primarily released from uranium-rich igneous rocks (e.g., granite) and sedimentary formations (e.g., phosphate rocks, shales) with higher uranium content. Limestone, although generally low in uranium content, can also emit radon in trace amounts^16,17^. Building materials, including concrete and gypsum wallboard, may also emit radon if they contain traces of uranium^18,19^. Because radon gas is significantly denser than air, once emitted, it tends to accumulate in the lowest areas of buildings (e.g., basements, below-ground spaces), although elevated radon levels can also be found in poorly ventilated ground-level rooms^20,21^.

### Factors modifying indoor radon concentrations

Radon concentration at a specific location is not a random occurrence but is determined by a complex interplay of geological, structural/architectural, and meteorological factors. These factors can either exacerbate or attenuate radon levels and ultimately influence the extent of indoor radon exposure.

#### Geological factors

Geologic formations with elevated uranium content (e.g., granites, shales, phosphate rocks) provide a higher potential for radon generation^16,17,22^. Additionally, the physical properties of soil, including its density, porosity, and moisture levels, are key determinants of radon migration from the subsurface to the atmosphere^17,22,23^. For example, clay-rich soils tend to inhibit radon diffusion and release, whereas sandy soils facilitate radon mobility. Moreover, geological discontinuities (e.g., faults and fractures) further enhance the transport of radon by providing pathways for upward migration of gas into the environment^17,24,25^.

#### Structural and architectural factors

The entry and accumulation of radon within buildings are strongly influenced by architectural and structural factors. The construction materials themselves may serve as sources of radon emissions. For example, stone foundations can emit higher levels of radon compared to foundations made of other materials.^21^ Older buildings that suffer from structural degradation and the presence of cracks typically exhibit higher radon concentrations due to an increased number of infiltration points^26^. Ventilation systems play a significant role in moderating indoor radon levels, and well-ventilated spaces dilute radon concentrations more effectively^21,26^. Furthermore, numerous other factors (as derived from census data) could contribute to indoor radon concentration, including the number of housing units, their occupancy status, structural attributes (e.g., the number of units in a structure), and type of heating fuel used^20,27^.

#### Meteorological factors

Meteorological conditions play an important role in fluctuating levels of indoor radon concentration. Temperature and pressure-driven flow affect radon’s movement from the ground into buildings. For example, lower indoor atmospheric pressure can increase radon entry, with higher temperature inside a household relative to outdoor resulting in pressure differentials that can facilitate the entry of radon into a structure^28^. Seasonal changes of temperature can further impact indoor radon levels, and concentrations are often higher during the winter than during the summer^29,30^. Furthermore, weather patterns, including precipitation, and moisture-related conditions can also change radon concentrations. Heavy rainfall can increase soil moisture and potentially reduce radon emissions, whereas dry conditions can allow for greater radon emissions from the dry soil^31,32^.

### Previous radon level prediction models

Previous studies have employed different modeling approaches to estimate radon concentrations across multiple geographic regions. The Environmental Protection Agency’s Radon Zone project classified counties based on geological and soil characteristics, offering a broad but useful categorization^33^. Subsequent studies have used more sophisticated statistical methods. Price et al.^34,35^ employed Bayesian models that incorporated geological data to improve county-level predictions of radon concentrations in Minnesota and mid-Atlantic states. Mose and Mushrush^23^ explored the correlation between soil radon levels, permeability, and indoor radon levels, emphasizing the complexity of radon entry into homes. Apte et al.^36^ employed mixed-effects regression models for predicting radon levels in New Hampshire, accounting for housing types and geological features. Similarly, Smith and Field^14^ developed a Bayesian hierarchical model to predict residential radon in Iowa by combining regional geological data and housing characteristics, and Casey et al.^37^ analyzed how geology, well water usage, and other factors influenced radon levels in Pennsylvania.

Recent studies have also applied machine learning techniques to more accurately predict radon levels. Kropat et al.^38^ and Nikkilä et al.^39^ used random forests (RFs) and other ensemble methods to predict radon concentrations in Switzerland and Finland, respectively, by incorporating detailed geological data. Dai et al.^40^ identified geological fault zones and housing characteristics as critical factors for radon risk in Georgia. Li et al.^41^ introduced an ensemble-based machine learning model that integrated multiple data types to predict monthly radon concentrations at the ZCTA level in the greater Boston area. These advanced approaches allow for the integration of environmental, architectural, and demographic factors, thereby enabling more comprehensive and nuanced radon risk assessments.

## Methods

This study employed machine learning techniques, specifically RF and quantile regression forest (QRF), to estimate the mean and quantiles of radon concentrations. We also used a volatility model framework and modeled the relative variability of radon at the ZCTA level. Residential radon values are from pre-mitigation indoor home radon tests. Predictive attributes include the physical characteristics known to affect radon exposure, and they were measured at the ZCTA level (elevation, soil, hydrologic, meteorological, and census data). Data were integrated to create predictive models that incorporate information from over 60 features related to residential radon exposure. These datasets were preprocessed and aggregated by using the H3 spatial indexing system, an open-source hierarchical hexagonal grid developed by Uber that partitions the Earth’s surface into hexagonal cells at multiple resolutions. In this study, we used level 8 H3 cells as a common spatial scale to align all datasets, using workflows developed for the Centralized Health and Environmental Repository (C-HER)^42,43^. The application of C-HER workflows enabled the harmonization of data across multiple spatial scales into a single reference scale. Detailed descriptions of the data processing methodologies for each dataset are provided in the associated methods white paper^44^. A concise overview of these methodologies is included in this paper for reference.

All maps in this manuscript were created by the authors in Python 3.11.14 (Python Software Foundation) using GeoPandas 1.1.1^45^ and Matplotlib 3.10.7^46^. ZCTA boundaries are based on the 2020 U.S. Census Bureau ZIP Code Tabulation Area shapefile^47^.

### Data processing

#### Residential radon values

Indoor radon test data collected from 2008 to 2021 were provided by the Pennsylvania Department of Environmental Protection (PA DEP) (*n* = 1,622,169).^48^ All of the available measures were included in the analysis. Pennsylvania was selected as the study area owing to the high prevalence of radon in the state. The dataset includes detailed information such as county, residential address postal code, building purpose, test floor level, and test date. The radon test data provided by PA DEP were address-masked for privacy, and only ZIP codes (later linked to ZCTAs) were available. The residential radon measures from 2008 to 2021 showed a 5.92 pCi/L average, a 2.60 pCi/L median, 1.40 pCi/L for the 25th percentile, and 5.40 pCi/L for the 75th percentile. Radon concentrations are expressed in picocuries per liter (pCi/L), which is a measure of how much radioactivity is present in a given volume of air. In simple terms, it reflects how many radioactive decays occur each second in a liter of air. One picocurie per liter corresponds to 0.037 becquerels per cubic meter (Bq/m³), the international (SI) unit for radioactivity. Higher values indicate a greater number of radioactive particles being released into the air, which in turn reflects higher potential exposure. Radon measurements in the dataset were obtained using charcoal canisters, alpha-track detectors, electret ion chambers, continuous monitors, and liquid scintillation methods. All measurement types were included in the analysis as reported, we did not attempt to calibrate results from different testing methods.

**Fig. 1:**
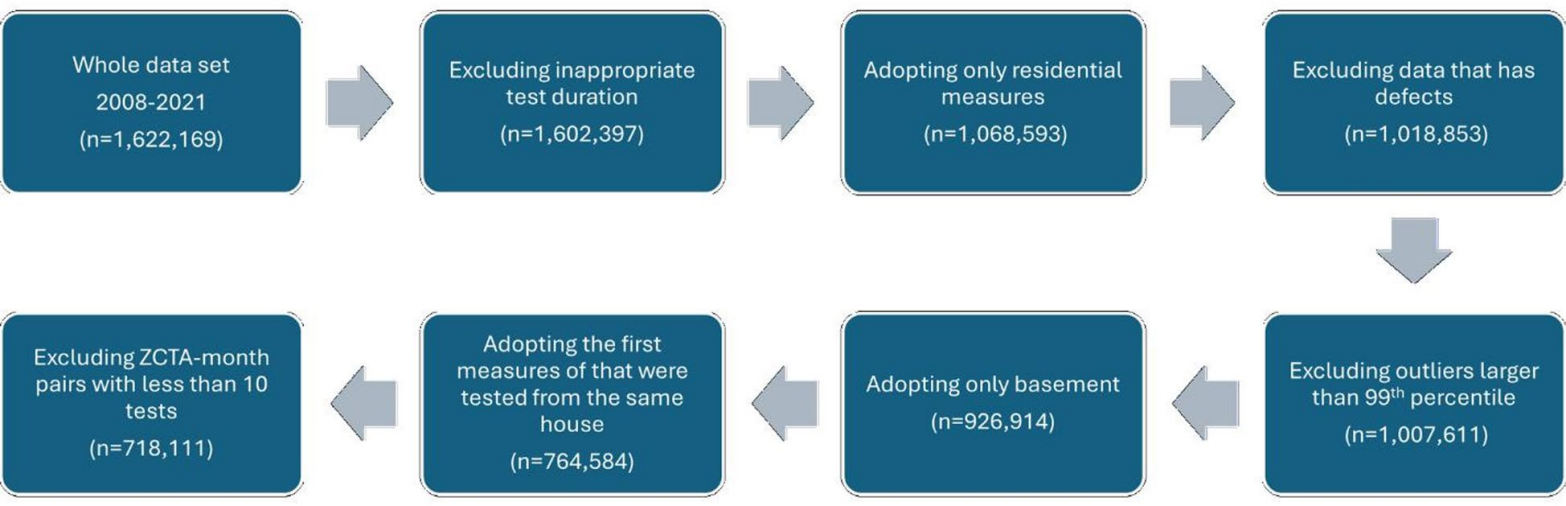
Process of the data selection and the number of measures after each step.

In this study, several exclusion criteria were applied to refine the dataset (Fig. 1). Tests with inappropriate durations (fewer than 2 days or more than 15 days, *n* = 9,772) were removed. Non-residential indoor radon tests were excluded (*n* = 533,804). Tests with out-of-range values, specifically those less than 0 or greater than 9,999, as well as tests lacking information on the test floor level, were also excluded (*n* = 49,740). Additionally, any test with a value exceeding the 99th percentile within a single zip code was excluded (*n* = 8,242). To address differences in indoor radon concentrations across floors, only measurements taken in basements, where radon levels are typically highest, were included (*n* = 80,697). For houses with multiple measurements across time, only the first recorded measurement was retained (*n* = 162,330) because subsequent measurements were likely taken post-mitigation.

Zip codes for radon test kit addresses were linked to Census ZCTAs by using a 2020 crosswalk file from the Uniform Data System Mapper^49^. For cases when no direct match was available, USPS zip code updates were applied.

To calculate ZCTA-level statistics, observations were clustered using ZCTA-month pairs for calculating averages or the coefficient of variations (CoVs) for a ZCTA, where the CoV is defined as the standard deviation divided by the mean. This also enabled us to account for the significant seasonal variability in radon levels^29,30^. Indoor radon concentrations are higher during winter months because structures typically use less ventilation, and heating a structure induces the stack effect^30^. The stack effect refers to the pressure difference that occurs when warm indoor air rises and escapes through the upper parts of a building, drawing in colder air from below. This airflow caused by the pressure difference can increase the entry of soil gases, including radon, into basements or ground-floor rooms.

We required at least 10 measurements per ZCTA-month pair to ensure the inclusion of statistically reliable averages and CoVs. ZCTA-month pairs with fewer than 10 measurements were excluded from the analysis (*n* = 46,473). After completing all data processing steps, the final dataset included 718,111 measurements across 1,542 ZCTAS from 2008 to 2021. This dataset was used to train and test predictive models.

**Fig. 2:**
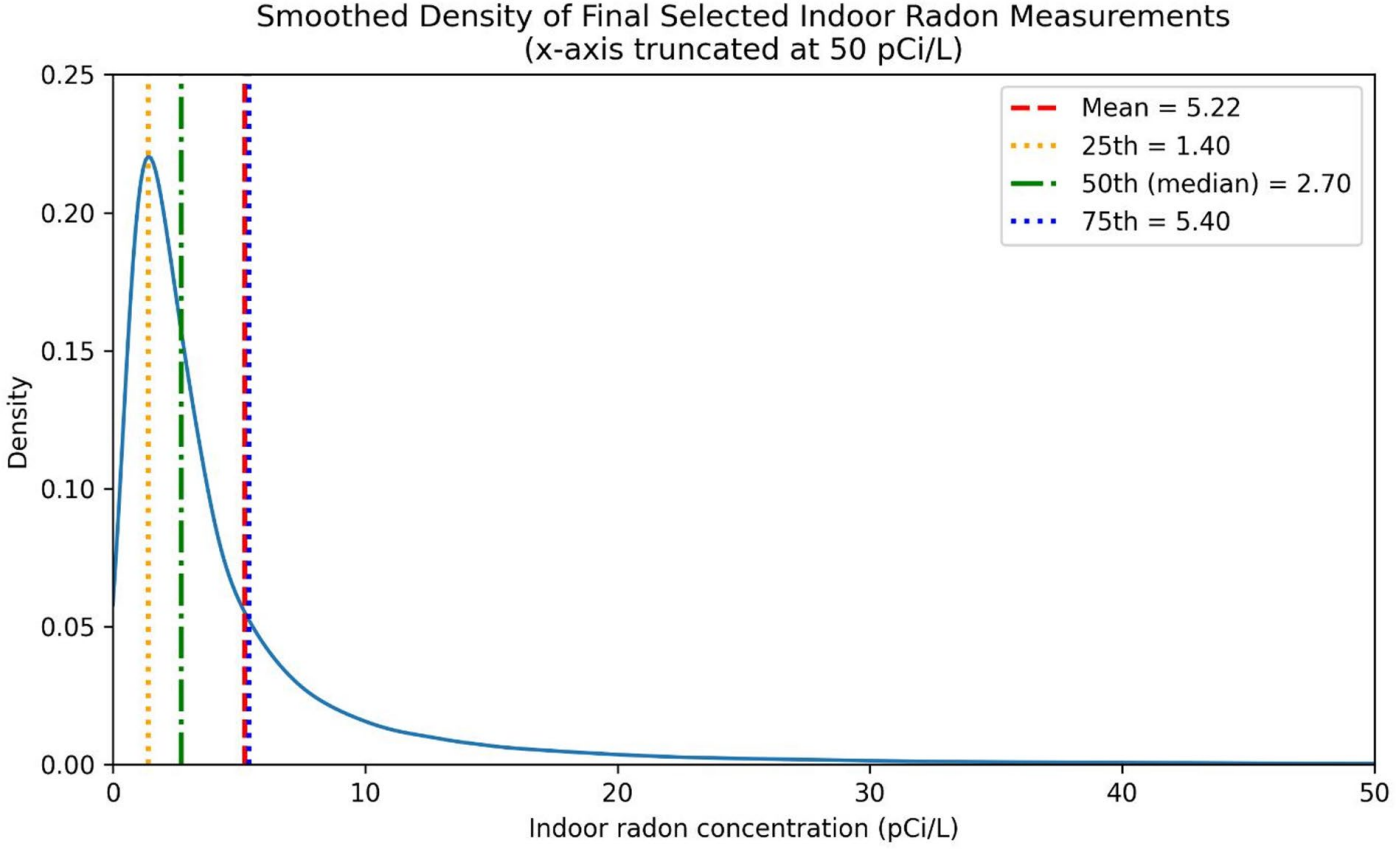
Process of the data selection and the number of measures after each step.

The processed residential radon measurements from 2008 to 2021 had an average concentration of 5.22 pCi/L, with a median of 2.70 pCi/L. The interquartile range spanned 1.40 pCi/L (25th percentile) to 5.40 pCi/L (75th percentile), highlighting significant variability in radon levels across Pennsylvania. (Fig. 2)

#### Independent variables

The elevation, soil, geochemical, hydrologic, and meteorological measures included in our model were reported across a range of spatial scales. Traditionally, to solve this issue, researchers aggregate measures to the spatial area of the dependent variable (i.e., ZCTA in our case). However, for measures such as soil data, for which there is a wide variability and more detailed information is available, this averaging results in less precise measures that decrease predictive accuracy. Additionally, in rural areas where housing may be sparsely spread across an area, averaging over a ZCTA incorporates information about soil characteristics that do not affect residential locations.

**Fig. 3:**
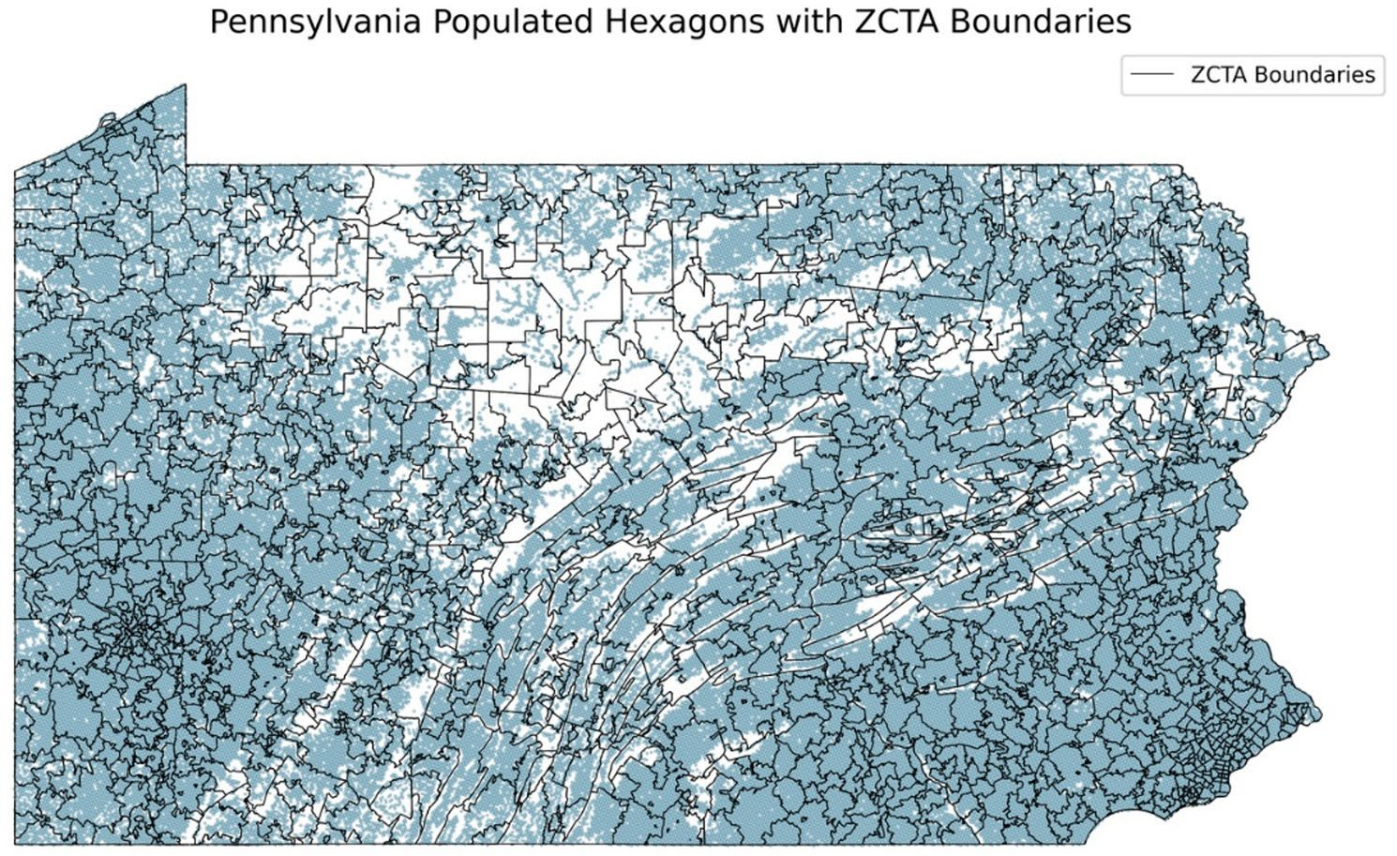
Populated hexagons in Pennsylvania and the boundaries of ZCTAs. Map was created in Python using GeoPandas^45^ and Matplotlib^46^ with ZCTA boundaries from the 2020 U.S. Census Bureau TIGER/Line shapefile^47^.

We developed a three-step process to solve these challenges^44^. First, all measures were spatially indexed using H3 hexagons. Second, we used LandScan population data^50^ to mask daily population data (aggregated day and night population) from 2018 to identify all H3 hexagons with an established population (Fig. 3). If a hexagon did not include a residential location, then the information from the hexagon was excluded from the aggregate statistics at the ZCTA level. Third, all data in H3 hexagons were converted to ZCTA (weighted average, standard deviation, and most frequent condition for categorical variables) by using areal interpolation. Due to unavailability of street addresses associated with radon measures, we could not assign measurements to exact point locations, and all environmental and housing covariates had to be constructed at the ZCTA level. To effectively manage large data volumes, a raster was conducted in overlapping tiles to ensure comprehensive coverage of all hexagons with relevant raster data. Census data were measured at the ZCTA level. However, the method of selecting a single most frequent category inevitably overlooks the heterogeneity of geological and geochemical conditions within a ZCTA. A single most frequent category cannot fully capture this heterogeneity. Therefore, while this aggregation method has limitations in that it may weaken the association between geological and geochemical covariates and radon and contribute to unexplained residual variability in radon levels, we adopted this approach due to limitations in the available data.

This study incorporated elevation data from the USGS’s Global Multi-Resolution Terrain Elevation Data 2010 (GMTED2010),^51^ which offers comprehensive global elevation information that is ideal for environmental and geological analyses. The GMTED2010 data for Pennsylvania was obtained in a 30 arc-second resolution and re-gridded into level-8 H3 hexagons using the geo_to_h3_aggregate() function from the h3pandas library,^43,52^ which calculated mean elevation for each hexagon based on raster pixel centroids. To address missing hexagons caused by grid misalignment, a ring smoothing technique was used to average values from adjacent hexagons to fill the gaps.

The soil data utilized in this study were extracted from the Gridded National Soil Survey Geographic Database (gNATSGO), which was provided by the USDA’s Natural Resources Conservation Service^53^. The gNATSGO dataset included detailed soil characteristics relevant for assessing radon emanation, transport, and accumulation. The analysis utilized 10-meter resolution state-level data in an ESRI file geodatabase format, and the data were processed using the ArcGIS Toolbox Soil Data Development Toolkit from the USGS. Soil maps were generated for each soil characteristic, representing the most frequent soil condition and the maximum available depth (200 cm). Individual rasters were reprojected to the EPSG:4326 coordinate reference system, and pixel values were extracted to construct a data frame that contained longitude and latitude. Zonal statistics for target hexagons were calculated using the geo_to_h3_aggregate() function from the h3pandas library.

This study incorporated geochemical data from the USGS Geochemical and Mineralogical Survey,^54^ and focused on uranium, potassium, and thorium concentrations across different soil depths (0–5 cm, A horizon, C horizon), where the A horizon represents the topsoil enriched with organic matter and the C horizon represents the substratum, an unhardened, largely unweathered layer that lies above bedrock. Because uranium is a direct source of radon, understanding its distribution is key for radon risk assessment. Geochemical data were processed into H3 hexagons to aggregate values at the ZCTA level, allowing for detailed spatial analysis. Categorical values from the GeoTIFF files were assigned using the geo_to_h3_aggregate() function to obtain the most frequent condition within ZCTAs, thereby ensuring consistency across hexagons and enhancing the accuracy of radon risk predictions.

Hydrologic data for this study were obtained from the USGS Hydrologic Landscape Regions, which provide detailed water-related landscape characteristics across the United States^55^. This dataset provides information on hydrological factors that affect radon transport through water and soil pathways. For the radon concentration prediction model, relevant hydrological variables were selected from an initial vector dataset at a 1 km × 1 km resolution. The data were converted into H3 level-8 hexagons by using the polyfill_resample() method from the h3pandas library to ensure consistency with other datasets.

The meteorological data used in the radon concentration prediction model were sourced from the Daymet dataset,^56^ which provides high-resolution climate information from across North America. Precipitation, snow water equivalent, temperature, and vapor pressure variables were because they may influence radon levels by affecting soil moisture, building ventilation patterns, and indoor-outdoor temperature gradients. Daily 1-km grids from Daymet were aggregated into monthly averages using numpy^57^ and then re-gridded. We converted all data to H3 level-8, a standardized hexagonal grid system used for mapping and spatial analysis. This conversion was performed with the area_interpolate() function, which implements areal interpolation methods from the PySAL Tobler spatial analysis library^58^. In practical terms, this method allows information from irregular geographic areas (such as ZIP codes or census tracts) to be translated into uniform hexagonal cells, making it easier to compare locations and analyze spatial patterns consistently.

The demographic and housing data used in this study were sourced from the American Community Survey (ACS) and Decennial Census (DEC) and accessed via the US Census Bureau’s API service. ^59,60^ These data were utilized to capture housing characteristics, which were hypothesized to be significant predictors of radon levels. Variables such as year of construction and primary heating fuel were collected from the 2000 DEC and the 2013–2020 5-year ACS estimates at the ZCTA-level resolution. A summary of selected variables and the rationale for their inclusion in the models are provided in Table 1.

**Table 1:** Variables included in the study for modeling radon level Estimation and the descriptions of their relevance and mechanisms by which they May influence radon levels.

Variable	Description
Bulk density	Reflects soil’s ability to permit radon gas movement; denser soils may reduce radon’s upward migration.
Percent clay, Sand, Silt	These factors affect soil permeability to radon gas, with coarser soils (higher sand content) generally allowing for easier radon passage.
Depth to soil restrictive layer	Indicates potential barriers to radon movement toward the surface.
Linear extensibility, Liquid Limit, Plasticity Index	Relate to soil’s expandability and water retention, impacting radon diffusion.
Surface texture	Influences the surface’s ability to release or trap radon gas.
Soil taxonomy classification	Provides insights into the soil’s overall characteristics that could affect radon emanation and transport.
Available water supply/Capacity	Water saturation levels can impact radon solubility and its movement through the soil.
Water content	Water content affects soil’s radon transmission properties as wetter soils may impede radon flow^22^,^28^.
Hydric rating by map unit	Identifies water saturation in soil. Moisture content can influence radon solubility and transport.
Hydrologic soil group	Classifies soils based on their drainage capacity, affecting radon’s upward movement from soil to indoor environments.
Soil moisture class/Subclass	Indicates moisture content of soil, which is essential for understanding radon transport dynamics in different soil conditions.
Soil temperature	Soil temperature can affect diffusion and permeability of radon.
Drainage class	Efficient drainage can reduce radon’s upward movement, making these variables critical in predicting radon levels.
Saturated hydraulic conductivity	Higher conductivity suggests easier movement of water and possibly of radon through the soil.
Organic matter	Influences soil structure and hence radon migration, with higher organic content potentially impeding radon movement.
Dwellings With/Without Basements	Indicates building characteristics that are directly related to potential indoor radon levels, as homes with basements are generally at higher risk.
Uranium content	Radon-222 is a decay product of uranium-238.
Thorium content	Radon-220 (or thoron) is a decay product of thorium-232.
Potassium content	Although potassium is not related to radon directly, it shows the characteristics of the soil or rock.
Clay content (10 Å, 14 Å, and Kaolinite)	Different types of clay content can affect radon’s retention and movement through soil, thereby influencing its diffusion and accumulation indoors.
Depth to bed rock	Depth to bedrock can determine how easily radon migrates from the subsurface to the surface, thereby impacting potential radon exposure levels in buildings.
Aquifer permeability class	Determines groundwater flow rates, which affect radon’s transport from soil to water sources and potentially into buildings.
Elevation	Influences atmospheric pressure variations, which can affect soil gas emissions, including radon.
Relief of watershed	Indicates topographical variations that can influence radon gas accumulation and dispersion patterns.
Percent flat land in watershed	Affects water drainage and soil gas movement, thereby impacting radon release.
Daily total precipitation	Impacts soil moisture levels, which can affect radon solubility and mobility through the soil.
Snow water equivalent	Reflects the amount of water contained in snowpack, which influences ground moisture and radon emission rates.
Daily minimum/Maximum 2-meter Air Temperature	Affects the thermal gradient between the ground and atmosphere, which influences radon diffusion. Also affects the ventilation habits that can affect accumulation of radon.
Vapor pressure	Vapor pressure can affect the moisture of soil, which can affect permeability of radon.
Occupancy status	Unoccupied houses may have higher radon concentrations because the ventilation systems may not operate regularly.
Year structure built	Older buildings may have more radon entry points due to structural degradation over time and might have different types of building materials.
House heating fuel	Different heating systems can alter indoor air pressure and flow, thereby influencing radon entry and distribution. Also, heating fuel itself can be a potential source of radon.

### Radon level estimation models

Although individual-level radon test results with zip codes were available, exact location information for each house tested was not provided. To protect privacy, the data provider masked the exact address of the test and released only the zip-codes. Although address masking was necessary for maintaining privacy, it reduced the accuracy of prediction algorithms, particularly in areas with high local variability. To address these limitations, a multi-model approach was employed to evaluate and compare the performance of various modeling strategies in the absence of precise location information. The RF algorithm^61^ was selected as the base model for all comparisons, and allowing us to account for complex interaction effects between variables in our model. Additionally, the QRF model, an extension of RF, was for the individual-level analysis. This approach enabled a more detailed assessment by estimating the conditional distribution of radon concentrations^62,63^. The ability to estimate the conditional distribution makes QRF particularly effective for assessing exposure risks by modeling quantiles of radon exposure rather than just the mean or median concentration levels.

For all models, the dependent and independent variables were measured at the ZCTA level because point-level information could not be assigned to individual radon tests. A comparison of three different models was used to provide a richer characterization of radon concentration risk within a ZCTA: (1) an Average Model, (2) a Relative Variability Model, and (3) an Individual QRF Model.

As the distribution of indoor radon concentrations was highly right-skewed, all prediction models were fit after log-transformation of radon concentration. This transformation reduces the influence of extremely high values and helps stabilize residual variance. For reporting and evaluation, model predictions were back-transformed to the original scale, and all performance metrics (RMSE, R², MAPE) were computed using observed and predicted radon concentrations in pCi/L.

Using multiple models enabled the quantification of model uncertainty in predicting indoor radon at the ZCTA-level. This ability, in turn, provides a framework for the interpretation of radon exposure levels in the absence of fine-scale spatial data.

#### Average model

The Average Model was designed to estimate the mean indoor radon concentration within each ZCTA, which is the standard approach for ecological radon risk assessment. Radon measures were averaged within each ZCTA-month pair. For variables other than the radon measure, numerical variables were averaged across the months, and categorical variables were selected with the most frequent value within each ZCTA-month pair. This method preserved seasonal information by grouping the dataset by both ZCTAs and months. The model fit for these models is generally calculated using the mean radon level for the ZCTA. However, ecological approaches that aggregate values with a geographic area ignore the underlying variability in the indoor radon exposure known to exist within a ZCTA. To illustrate the limitations of an ecological approach, we recalculated the model fit using the individual radon tests as the observed value and the prediction from the Average Model.

#### Relative variability model

In the second model, we predicted the variability of indoor radon exposure in a ZCTA by using RF to identify characteristics of ZCTAs with a high variability of indoor radon exposure. In this model, the CoV was used for radon measurements, defined as the ratio of the standard deviation to the mean. We hypothesized that variability in geographic, meteorological, and housing characteristics would be associated with variability in indoor radon exposure within a ZCTA and therefore used the variability of these factors to predict the CoV of radon. For numerical predictors, we calculated the standard deviation across H3 level-8 hexagons within each ZCTA; for categorical predictors, we used Shannon entropy to quantify within-ZCTA heterogeneity, computed as Eq. (1)

1 $$\:\mathrm{H}=-\:\sum\:_{k=1}^{K}{p}_{k}{\mathrm{l}\mathrm{o}\mathrm{g}}_{2}\left({p}_{k}\right)$$

where pk is the proportion of H3 cells in each category.

Owing to the lack of within-ZCTA variability information from ACS and DEC, we could not include measures of variation for fuel type, age of structure, or occupancy status. This approach enabled the quantification of uncertainty in predicted averages based on the variability of the input data. The Relative Variability Model complemented the Average Model by identifying areas with highly heterogeneous indoor radon exposure—a phenomenon that aggregate means alone could not capture.

#### Individual QRF model

The QRF algorithm extends RF by estimating the conditional distribution of the outcome rather than only its mean. This nonparametric machine learning method allows direct prediction of any quantile of radon concentrations, providing a way to evaluate both the range and uncertainty of exposure. Such an approach is particularly valuable for risk assessment and public health planning, because upper-tail concentrations often drive mitigation decisions and health risks.

In previous studies, QRF has been applied to various environmental datasets after the introduction of the algorithm by Meinshausen^63^(2006). Work by Vaysse and Lagacherie^64^(2017) and Maxwell^65^ (2021) applied QRF to geological studies. These studies highlighted the utility of QRF in capturing the distribution and the variabilities of target variables, making it an appropriate choice for radon prediction in this study.

The quantile-forest python package was used to implement QRF^62^. The model was trained on the same set of geological, meteorological, and building-specific predictors used in the RF-based models. For each ZCTA, the dependent variables were the percentiles of the individual level radon test results within a ZCTA. The independent variables are the population-masked averages or most frequent values at the ZCTA level, same as Average Model and the population-masked standard deviation or the entropy of the variables at the ZCTA level same as the Relative Variability Model. This model will allow us to identify ZCTAs that may have high levels of indoor radon exposure. Owing to the computational complexity of QRF, training and prediction required substantially more time than RF; we therefore used high-performance computing resources in the Compute and Data Environment for Science (CADES) and Andes at Oak Ridge National Laboratory to efficiently carry out the analysis.

#### Model evaluation metrics

The prediction performance of the RF and QRF models was evaluated by using a combination of metrics to assess both predictive accuracy and model interpretability.

##### Predictive accuracy metrics

For the predictive accuracy metrics, root mean square error (RMSE), R-squared (R²), and mean absolute percentage error (MAPE) were used. RMSE quantifies the average magnitude of prediction errors, providing a measure of overall accuracy. MAPE measures the average percentage deviation between predicted and actual radon concentrations, offering an intuitive understanding of prediction error magnitude. As RMSE squares the residuals, it is particularly sensitive to outliers and large prediction errors. In our data, where radon concentrations are highly skewed with a long upper tail, RMSE primarily reflects model performance for homes with very high radon levels. MAPE also has limitations because it divides the absolute error by the observed value, predictions for observations with very low true radon concentrations can yield very large or unstable percentage errors, even when the absolute error is small. We therefore interpreted RMSE together with R² and MAPE.

To account for potential spatial autocorrelation within ZCTAs, grouped 5-fold cross-validation (CV) was implemented, in which folds were created based on ZCTA groupings.

##### Permutation feature importance and partial dependence plots

Permutation feature importance and partial dependence plots (PDPs) were analyzed to identify important features in each model. Permutation importance quantifies the reduction in model performance when the values of a feature are randomly shuffled, thereby identifying the features that are most critical for capturing the radon levels. Permutation feature importance was calculated as the mean decrease in out-of-sample R².

To address the issue of highly correlated variables that dilute feature importance, only one variable from each group of highly correlated variables (absolute correlation coefficient > 0.85) was included in the model. Table 2 presents the groups of variables with absolute correlation coefficients that exceed 0.85. Additionally, variables such as hydrologic soil group and drainage class, which were already represented in aquifer permeability, were excluded to avoid redundancy.

**Table 2:** Groups of highly correlated variables (|r| > 0.85). Bolded variables are used in the model to analyze the permutation importance.

Group	Variables
1	‘Minimum elevation in watershed,’ ‘**Elevation’**
2	‘Available Water Capacity WTA, 0 to 200 cm,’ ‘**Available Water Storage WTA**,** 0 to 200 cm**,’ ‘Available Water Supply, 0 to 25 cm,’ ‘Available Water Supply, 0 to 50 cm,’ ‘Available Water Supply, 0 to 150 cm,’ ‘Available Water Supply, 0 to 100 cm,’ ‘Water Content, 15 Bar WTA, 0 to 200 cm,’ ‘Water Content, One-Third Bar WTA, 0 to 200 cm’
3	‘Percent Clay WTA, 0 to 200 cm,’ ‘Linear Extensibility WTA, 0 to 200 cm,’ ‘Liquid Limit WTA, 0 to 200 cm,’ ‘Linear Extensibility WTA, 0 to 200 cm,’ ‘**Plasticity Index WTA**,** 0 to 200 cm**,’ ‘Percent Silt WTA, 0 to 200 cm’
4	‘Saturated Hydraulic Conductivity (Ksat) WTA, 0 to 200 cm,’ ‘**Saturated Hydraulic Conductivity (Ksat)**, **Standard Classes WTA**, **0 to 200 cm**’
5	**‘Percent flat land (slope less than 1%) in watershed lowland**,’ ‘Total percent flat land (slope less than 1%) in watershed’
6	‘**Maximum air temperature**,’ ‘Minimum air temperature,’ ‘Water vapor pressure’
7	‘**Estimate Total Utility Gas**,’ ‘Estimate total fuel oil, kerosene, etc.’
8	‘Potassium content in A horizon,’ ‘**Potassium content in 0 to 5 cm depth**’
9	‘Thorium content in A horizon,’ ‘**Thorium content in 0 to 5 cm depth**’
10	‘Uranium content in A horizon,’ ‘**Uranium content in 0 to 5 cm depth**’

PDPs were generated to visualize the marginal effects of individual features on the predicted radon concentrations. PDPs helped illustrate the relationship between each feature and the model’s predictions, revealing whether the relationship was linear, monotonic, or more complex. This visual analysis complemented the permutation feature importance metrics, offering a more intuitive interpretation of the model’s behavior.

## Results and discussion

The Average Model test was conducted by applying 5 iterations of the 5-fold CV with ZCTA as the grouping variable and then without grouping variables. The exercise was conducted again to test the radon-level prediction data for individual houses when using the average model (Table 3). The final analytic dataset contained 718,111 residential basement radon measurements corresponding to 865 ZCTAs across Pennsylvania. For the ungrouped 5-fold cross-validation, approximately 80% of observations (143,622 − 143,623) were used for training and 20% (692–693) for testing in each fold. In the ZCTA-grouped 5-fold cross-validation, each fold included 692–693 ZCTAs in the training set and 173–174 ZCTAs in the test set.

**Table 3:** Metrics and their standard deviation of average model tested with average and with individual data.

	Tested with average data		Tested with individual data	
	5-fold CV	Group 5-fold (ZCTA) CV	5-fold CV	Group 5-fold (ZCTA) CV
RMSE (pCi/L)	2.67 (0.19)	3.17 (0.14)	7.80 (0.15)	7.86 (0.30)
R^2^	0.67 (0.022)	0.53 (0.021)	0.12 (0.0020)	0.10 (0.0079)
MAPE (%)	20.68 (0.42)	27.71 (0.81)	166 (1.59)	167 (2.70)

The metrics indicated what we consider fair predictive performance for the average radon level in the community: the model explained 67% of the variance in mean radon concentrations in random 5-fold CV and 53% in ZCTA-grouped CV, with RMSE values of 2.67–3.17 pCi/L and MAPE of 21–28%. In other words, predicted ZCTA-level mean radon concentrations differed from the observed means by roughly one quarter on average, which we consider acceptable for ecological, community-level risk assessment. In the context of prediction models, R² in the range of ~ 0.5–0.7 and MAPE around 20–30% are generally interpreted as acceptable for population-level risk assessment^66,67^. In contrast, when the same model was evaluated against individual household measurements, performance deteriorated sharply. These results suggest significant variability in radon levels within each ZCTA, so that the use of the prediction of the average will not reflect the characteristics of individual houses. This limitation poses challenges from a public health perspective because some houses with high radon levels may remain untested due to low exposure levels in the surrounding areas, leading to gaps in risk identification and mitigation efforts. (line 424)

**Fig. 4:**
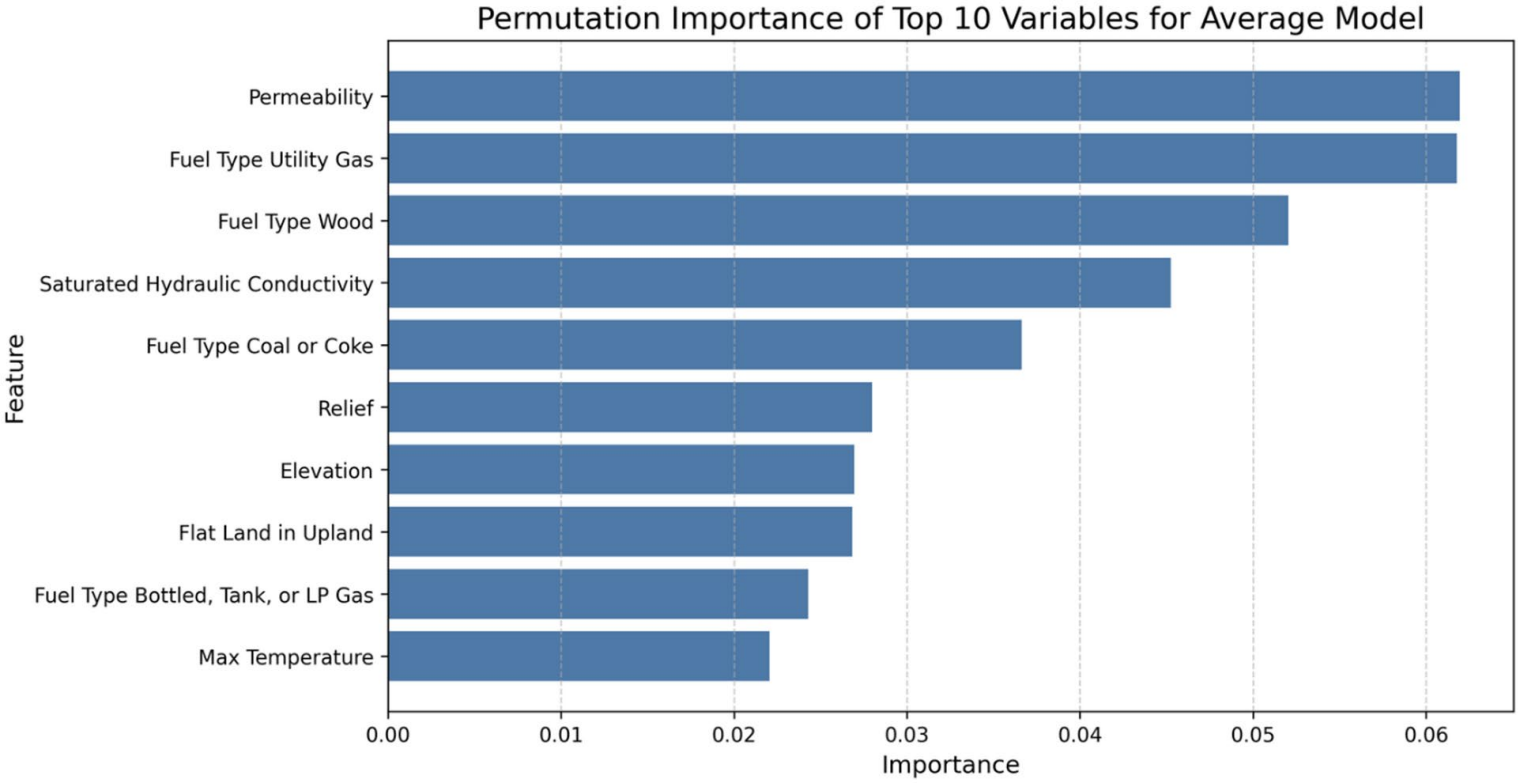
Permutation feature importance of the top-20 variables for the Average Model.

Permutation feature importance was assessed to identify the variables from the dataset that significantly affected average radon concentration predictions (Fig. 4). Permutation importance was computed as the mean decrease in out-of-sample R² when the values of a given predictor were randomly permuted, with larger values indicating stronger contributions to model performance.

**Fig. 5:**
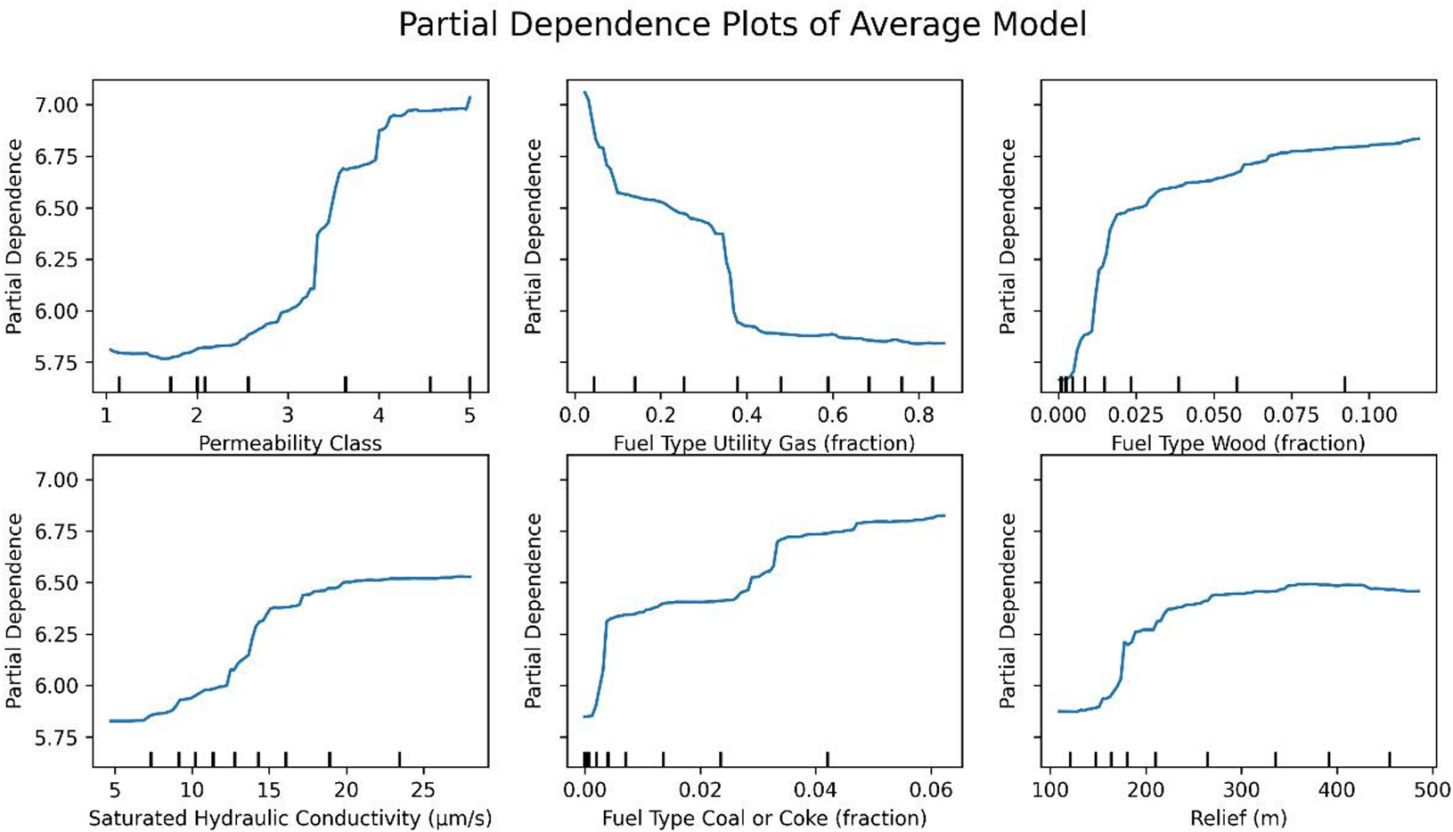
Partial dependence plots (PDPs) of the featured variables for the Average Model (top-6 importance non-categorical features, all variables are scaled).

To examine the impact of specific predictors on radon levels, PDPs were generated for the most influential non-categorical variables (Fig. 5). For each continuous predictor, we computed partial dependence plots (PDPs) to visualize its marginal association with predicted radon levels. For a given predictor, the x-axis shows the value of that predictor in its original units, and the y-axis shows the model-predicted average indoor radon concentration (pCi/L), obtained by averaging over the observed distribution of all other variables. A flat line indicates little association, whereas steeper or nonlinear patterns indicate stronger or nonlinear effects of that predictor on predicted radon levels.

Permutation importance revealed that permeability, house heating fuel type, and hydraulic conductivity had the largest effects on determining the average radon level.

The partial dependence of permeability suggests that increasing soil permeability or saturated hydraulic conductivity consistently results in higher average radon levels. Notable patterns are also observed in the PDPs for fuel types, where areas with higher usage of wood, coal, or coke as the primary fuel source exhibit elevated radon concentrations. In contrast, regions that use utility-provided gas heat as the main fuel type show lower radon levels. The partial dependence of relief indicates sharp increases in radon levels at lower relief values. Higher relief values have a diminishing effect beyond a certain point.

**Fig. 6:**
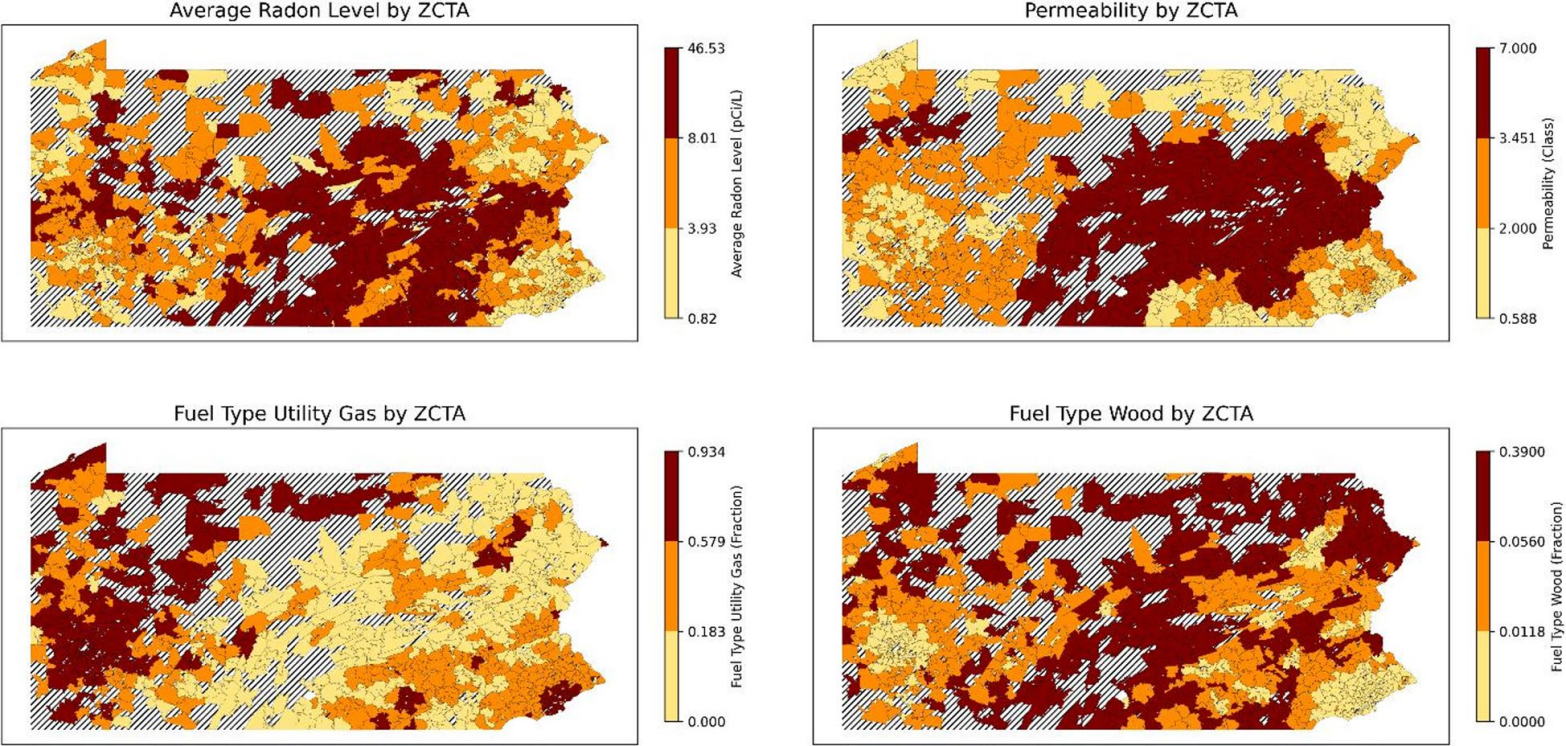
Actual average and three most important variables of January by ZCTA (and ZCTAs where more than 3 measures exist). Striped areas indicate ZCTAs where data is not available. Maps were created in Python using GeoPandas^45^ and Matplotlib^46^ with ZCTA boundaries from the 2020 U.S. Census Bureau TIGER/Line shapefile^47^.

Figure 6 illustrates the maps of the actual average radon levels alongside the averages of key variables by ZCTAs across Pennsylvania. The spatial distribution of average radon concentrations exhibited a notable resemblance to the permeability map. However, the map that shows utility gas as the primary fuel type indicates that urban areas generally have higher utility gas fuel usage and rural areas generally have higher wood fuel usage. In this study, we are unable to determine if fuel type directly influences radon concentrations or whether it serves as an indirect proxy for distinguishing urban from rural areas. Further analysis is warranted to discern the true relationship between fuel type and radon levels.

The Average Model effectively predicted radon levels at the ZCTA level, providing a more refined analysis compared to county-level estimates. Offering predictions at the ZCTA level enabled a more detailed assessment of radon exposure risk at a smaller geographic scale. Given the significant variability in radon levels even within small areas, such granular predictions are crucial for accurate risk analysis and targeted public health interventions.

The Average Model had a notable limitation in that it lacked detailed information on the variability of radon exposure within an area because all data were aggregated. As a result, the Average Model has a fair prediction accuracy for the radon levels of the community, but detailed predictions of radon levels for each house are not achievable. To address this, we used a Relative Variability Model for uncertainty quantification, enabling the identification of factors that predict high variability in ZCTA level measures.

As with the Average Model, the Relative Variability Model test was conducted by applying 5 iterations of the 5-fold CV, both with ZCTA as the grouping variable and without grouping variables (Table 4).

**Table 4:** Metrics and their standard deviation of the relative variability Model.

	5-fold CV	Group 5-fold (ZCTA) CV
RMSE	0.23 (0.0055)	0.27 (0.0059)
R^2^	0.46 (0.014)	0.24 (0.094)
MAPE (%)	16.68 (0.38)	20.76 (0.77)

**Fig. 7:**
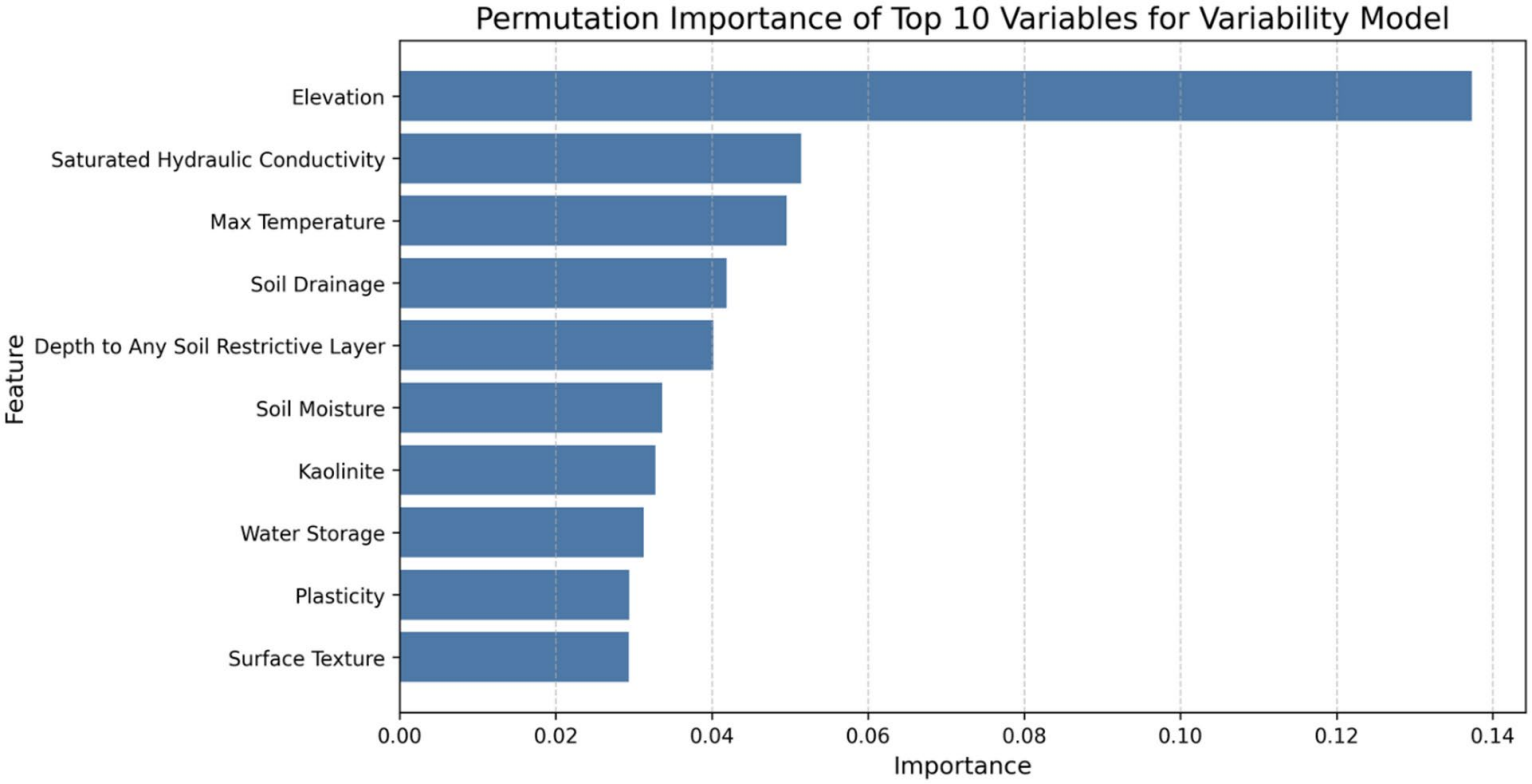
Permutation feature importance of top-10 variables for relative variability model.

Permutation feature importances and PDPs were evaluated with the Relative Variability Model to identify and illustrate the most influential factors that affect radon concentration variability, as shown in Fig. 7.

**Fig. 8:**
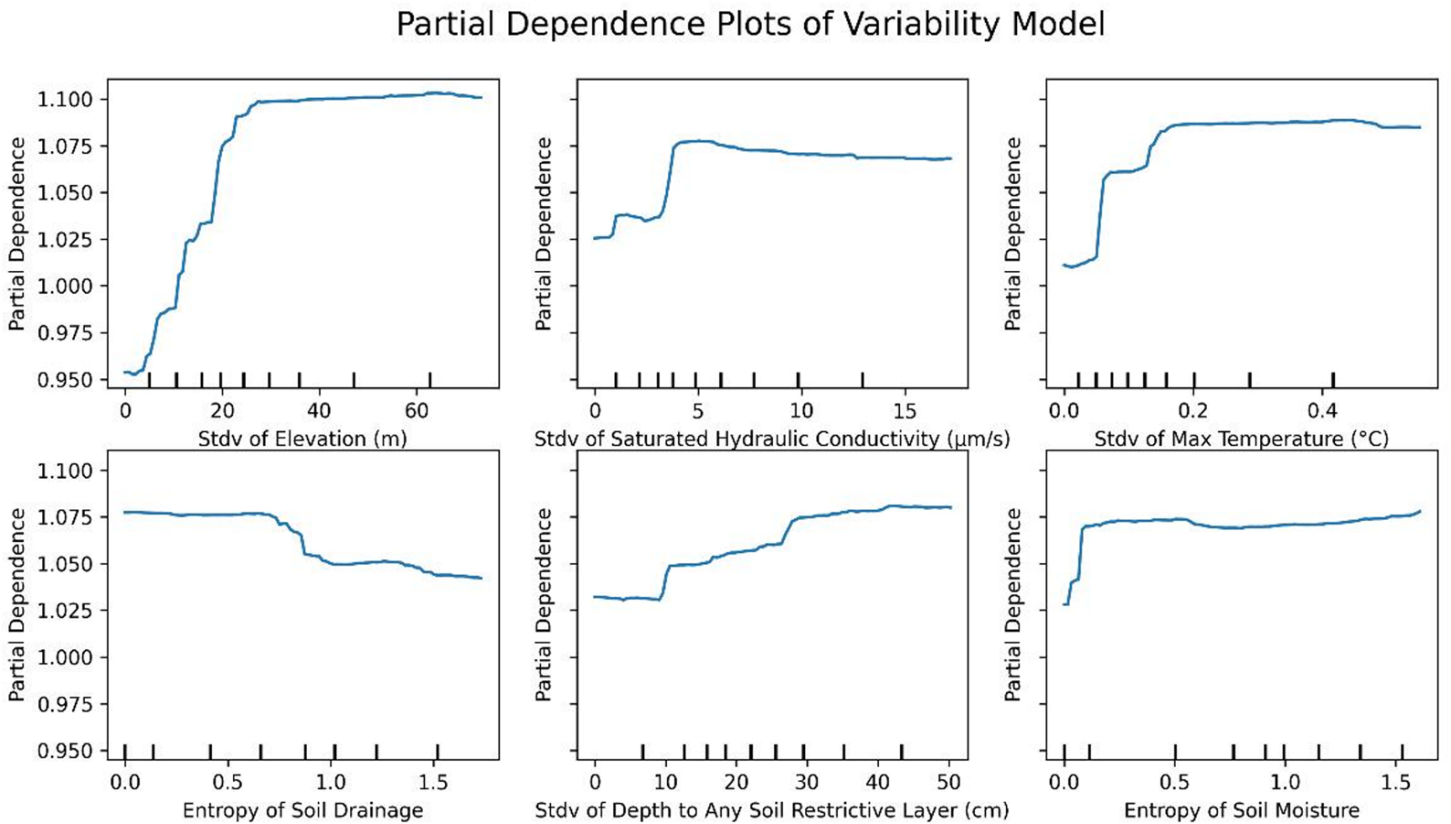
PDPs of the featured variables for the variability model (top-6 importance features).

Although permeability and fuel type were identified as the most important variables that influence average radon levels, the factors that predict variability in radon levels are different (Fig. 8). We find that ZCTAs with large variability in elevation, saturated hydraulic conductivity, temperature, soil drainage, depth to restrictive soil layers, and soil moisture, as well as variability in kaolinite content, available water storage, plasticity, and surface texture, exhibit high levels of variability, or uncertainty, around average radon levels. This discrepancy is not unexpected, as the determinants of the mean radon level are not identical to the determinants of within-ZCTA variability.

High permeability systematically increases indoor radon concentrations across a given ZCTA, potentially raising average concentrations even in areas with relatively uniform permeability. In such environments, while the average radon concentration may be high, the variability in radon measurements within that ZCTA may not be large as the geological conditions are fairly uniform. Furthermore, even if variations in permeability exist, if the degree of variation of radon from variation of permeability is minimal due to generally high or low permeability in the area, high variability of radon may not be observed.

Therefore, it is reasonable that predictor variables strongly correlated with average radon concentrations do not necessarily have the greatest influence on radon variability within the same area. The average model and the variability model capture complementary aspects of radon risk. The former reflects the general level of exposure, while the latter indicates the degree of heterogeneity and uncertainty of exposure within the community.

Partial dependence analysis of the standard deviation of elevation, hydraulic conductivity, maximum temperature, and soil moisture indicated that the variability of local radon concentrations initially increased with greater variation of these variables. However, this was non-linear, with the increases plateauing beyond a certain point. In contrast, uncertainty of radon continued to increase gradually with greater variability in soil depth to the restrictive soil level. Conversely, as the variability in soil drainage increased, the uncertainty in an average radon level showed a decrease. This counterintuitive result requires more research; however, it may be related to urbanization or differences in building materials/structures.

**Fig. 9:**
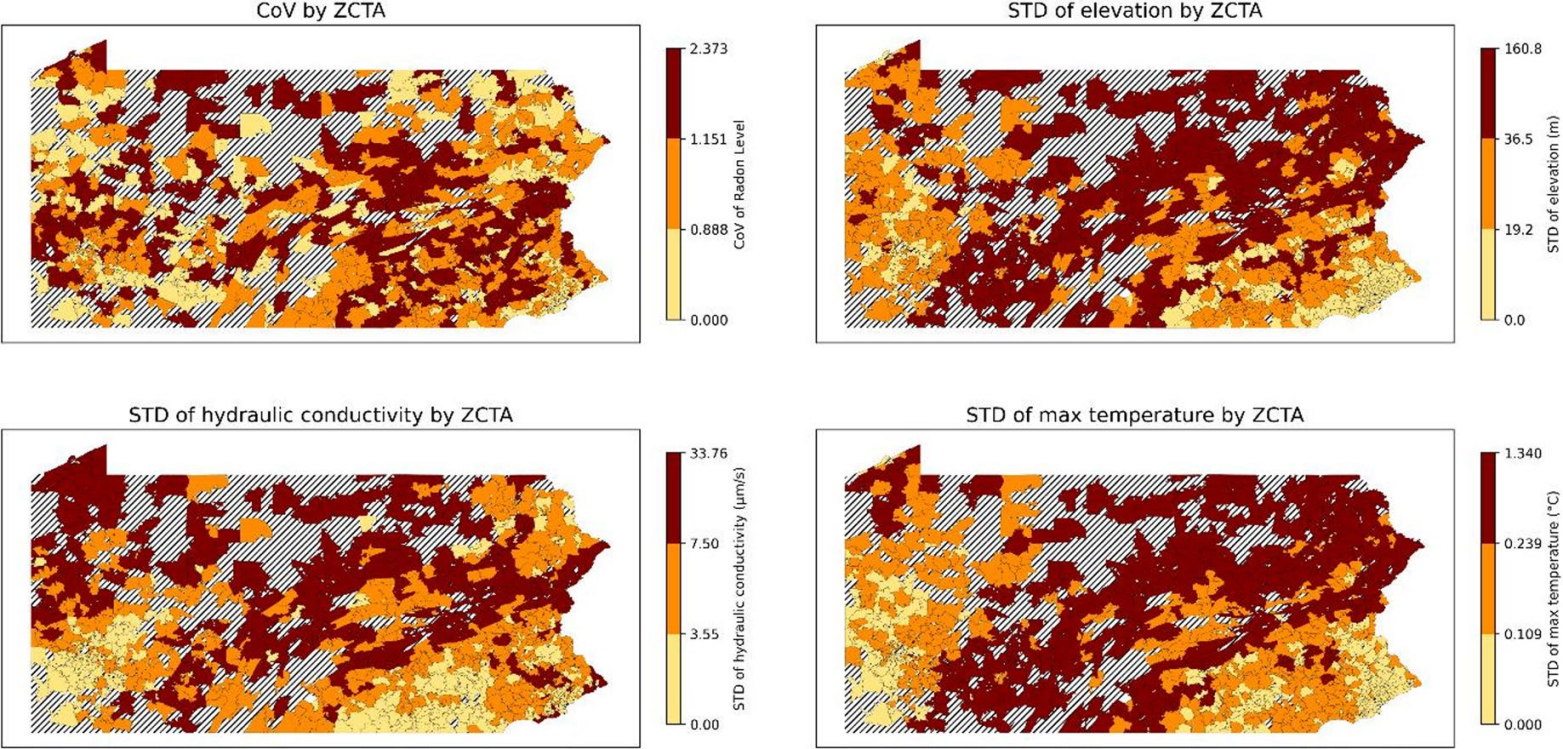
Actual coefficient of variance (CoV) and the standard deviations (STDs) of the top-3 most important variables for January by ZCTA (ZCTAs where more than 3 measures exist). Striped areas indicate ZCTAs where data is not available. Maps were created in Python using GeoPandas^45^ and Matplotlib^46^ with ZCTA boundaries from the 2020 U.S. Census Bureau TIGER/Line shapefile.^47^.

Figure 9 shows the CoV of radon levels by ZCTA and the standard deviations of key variables by ZCTAs across Pennsylvania. The correlation of the CoVs of radon measures to the standard deviations of variables are not as high as the mapped average levels.

The Relative Variability Model added value by quantifying the range and distribution of radon levels, highlighting areas with significant fluctuations and variabilities in radon concentrations. This model can be used to identify the characteristics of the area that has a higher chance of possessing the house with exceeding the action level.

**Fig. 10:**
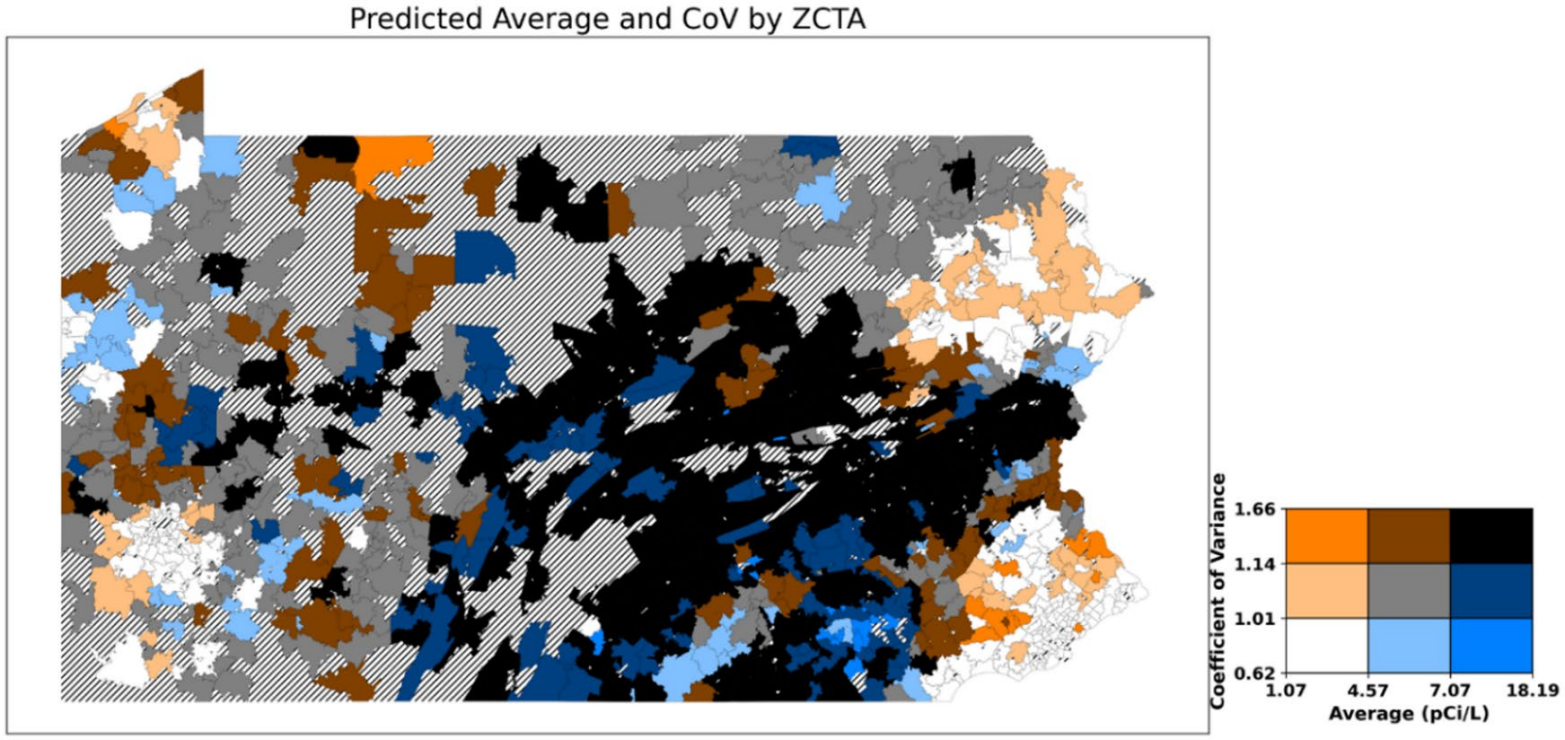
Predicted average and CoVs of radon levels in Pennsylvania for January by ZCTAs (ZCTAs where more than 3 measures exist). Striped areas indicate ZCTAs where data is not available. Map was created in Python using GeoPandas^45^ and Matplotlib^46^ with ZCTA boundaries from the 2020 U.S. Census Bureau TIGER/Line shapefile^47^.

Figure 10 illustrates how areas with high average radon concentrations can also have high variability of radon concentrations. Overall, the darker-colored areas on this map represent regions where testing for indoor radon exposure should be prioritized because there are both high average levels and high variability. The specific colors (ranging from shades of red to shades of blue) illustrate the characteristics of radon distribution in these areas. Regions shaded in red indicate areas with high variability.

Many areas exhibited similar patterns between the predicted average radon levels and the CoV of radon. However, some ZCTAs displayed distinct patterns of variability at the ZCTA level, even when their average radon levels were comparable. To illustrate this, we selected six example ZCTAs and refer to them with anonymized labels A–F. For example, ZCTA A (average: 10.58 pCi/L, CoV: 1.39), B (average: 9.80 pCi/L, CoV: 1.43), and C (average: 8.95 pCi/L, CoV: 1.43) had high radon levels and high CoV in the month of January for the 2008–2021 data. January. ZCTA D (average: 10.84 pCi/L, CoV: 0.88), E (average: 10.86 pCi/L, CoV: 0.94), and F (average: 8.99 pCi/L, CoV: 0.90) showed high average radon levels but showed low CoVs of radon in January.

Table 5 compares the standard deviation of key variables that have the highest importance in the Relative Variability Model. The standard deviation of elevation, hydraulic conductivity, and maximum temperature is much higher for the high variability groups than for the low variability groups. This result shows that, although the average level of radon is similar to other areas, the variability of the radon level itself can be different if the variability of the variables (e.g., elevation, hydraulic conductivity, maximum temperature) differs.

**Table 5:** Key variability for ZCTAs with high radon. High vs. low variability groups in January.

	ZCTA	Standard deviation of elevation (m)	Standard deviation of hydraulic conductivity (µm/s)	Standard deviation of maximum temperature (°C)
High average and high variability	A	70.63765	12.27862	0.635485
	B	32.44647	6.901275	0.22935
	C	29.22913	5.436235	0.184695
High average and low variability	D	7.444461	0.842725	0.035577
	E	3.86221	0.415112	0.010676
	F	12.89145	2.60695	0.05758

Some of the ZCTAs showed high variability of radon even though they had below-average radon levels. ZCTA G (average: 3.55 pCi/L, CoV: 1.36) and H (average: 3.60 pCi/L, CoV: 1.39) showed relatively low radon levels but high radon variability. H showed high variability in elevation (61.86 m), and G showed low variability in elevation and hydraulic conductivity.

The Average Model and the Relative Variability Model do not require significant computational resources and are relatively simple models for predicting average radon levels and variability. However, they cannot capture the full spectrum of information available in the dataset. To provide a more detailed characterization of the distribution of indoor radon exposure within a ZCTA, a QRF Model was used to predict radon concentrations across different quantiles, thereby providing a comprehensive view of radon exposure risks. The QRF model implemented using the quantile-forest library extends the capabilities of traditional RF models by estimating the conditional distribution of radon levels rather than focusing solely on mean predictions. This method allows for a detailed assessment of radon risk by predicting a range of possible outcomes at various quantile levels within each ZCTA.

RMSE, R2, and MAPE for the 50th, 75th, and 90th percentiles were used to evaluate the performance of the QRF model (Table 6). The evaluation was conducted with a grouped 5-fold CV by using ZCTA as the group. Based on the same reasoning as averaging ZCTA-month pairs for the Average Model, each predicted quantile value was tested with the actual quantile value of each ZCTA-month pair. To estimate and compare the estimated 90th percentile value with the measured 90th percentile value, the model used a dataset in which there were at least 10 measures for each ZCTA-month pair.

**Table 6:** Metrics and standard deviation of individual quantile regression forest (QRF) model.

	50th		75th		90th	
	5-fold CV	Group 5-fold (ZCTA) CV	5-fold CV	Group 5-fold (ZCTA) CV	5-fold CV	Group 5-fold (ZCTA) CV
RMSE (pCi/L)	1.65 (0.020)	2.01 (0.13)	3.65 (0.037)	4.51 (0.20)	7.01 (0.069)	8.64 (0.66)
R^2^	0.54 (0.0096)	0.32 (0.023)	0.63 (0.0065)	0.44 (0.023)	0.67 (0.0060)	0.50 (0.039)
MAPE (%)	18.90 (0.17)	24.86 (1.00)	22.05 (0.26)	31.10 (1.33)	30.01 (0.49)	44.07 (2.36)

Under standard 5-fold cross-validation, R² values were 0.54, 0.63, and 0.67 for the 50th, 75th, and 90th percentiles, respectively, with corresponding RMSE values of 1.65, 3.65, and 7.01 pCi/L. The MAPE ranged from about 19% at the median to 30% at the 90th percentile, indicating that relative error increases when predicting higher radon concentrations. When ZCTA-grouped 5-fold cross-validation was used, performance decreased (R² = 0.32–0.50 and higher RMSE/MAPE), reflecting the added difficulty of predicting radon distributions in ZCTAs that were not represented in the training set. Together, these results suggest that the QRF model provides reasonably accurate estimates of the distribution of radon concentrations within ZCTAs, while still leaving substantial uncertainty for extreme concentrations.

**Fig. 11:**
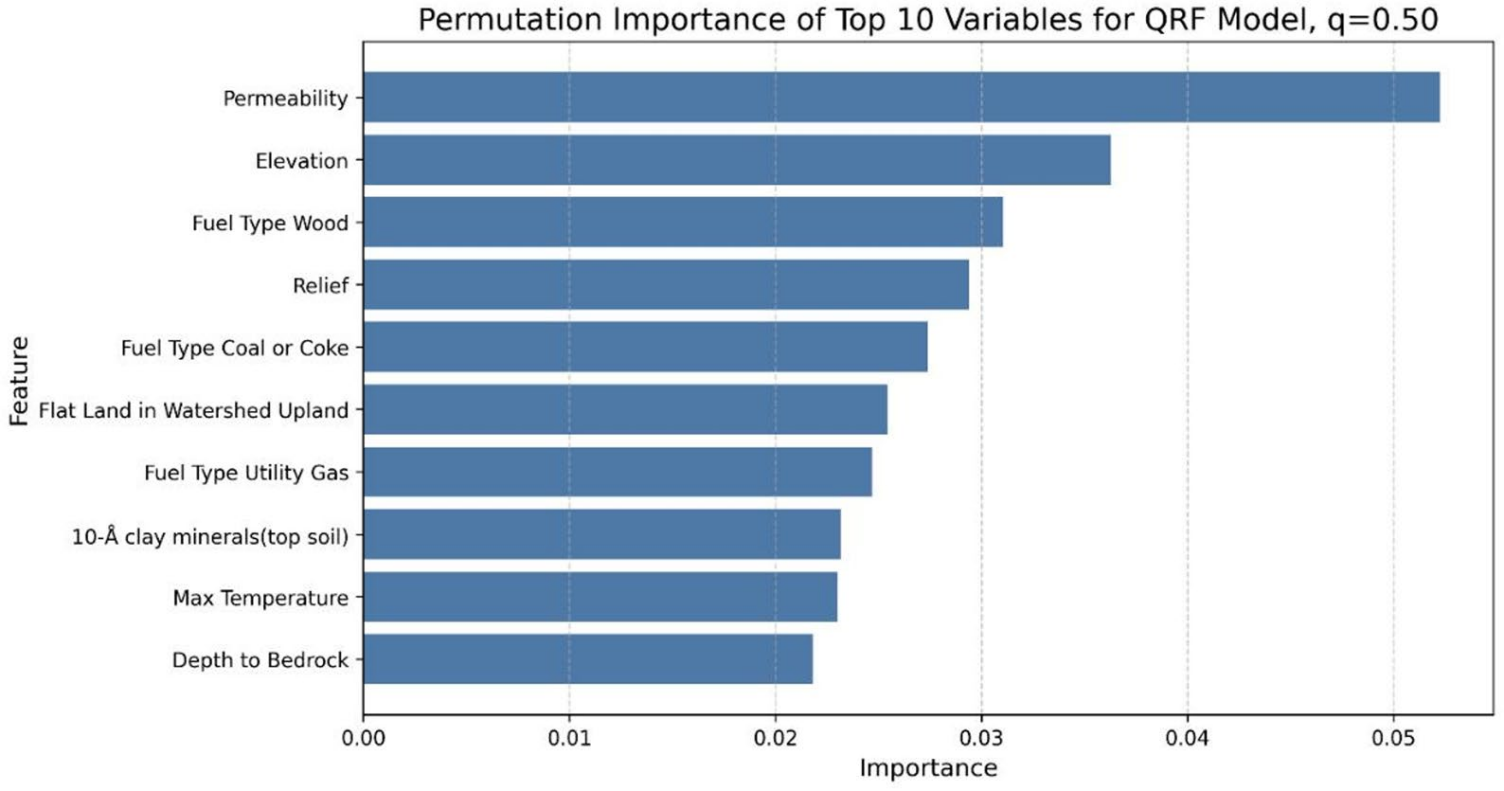
Permutation feature importance of top-10 variables for 50th percentiles of individual a quantile regression forest (QRF) model.

**Fig. 12:**
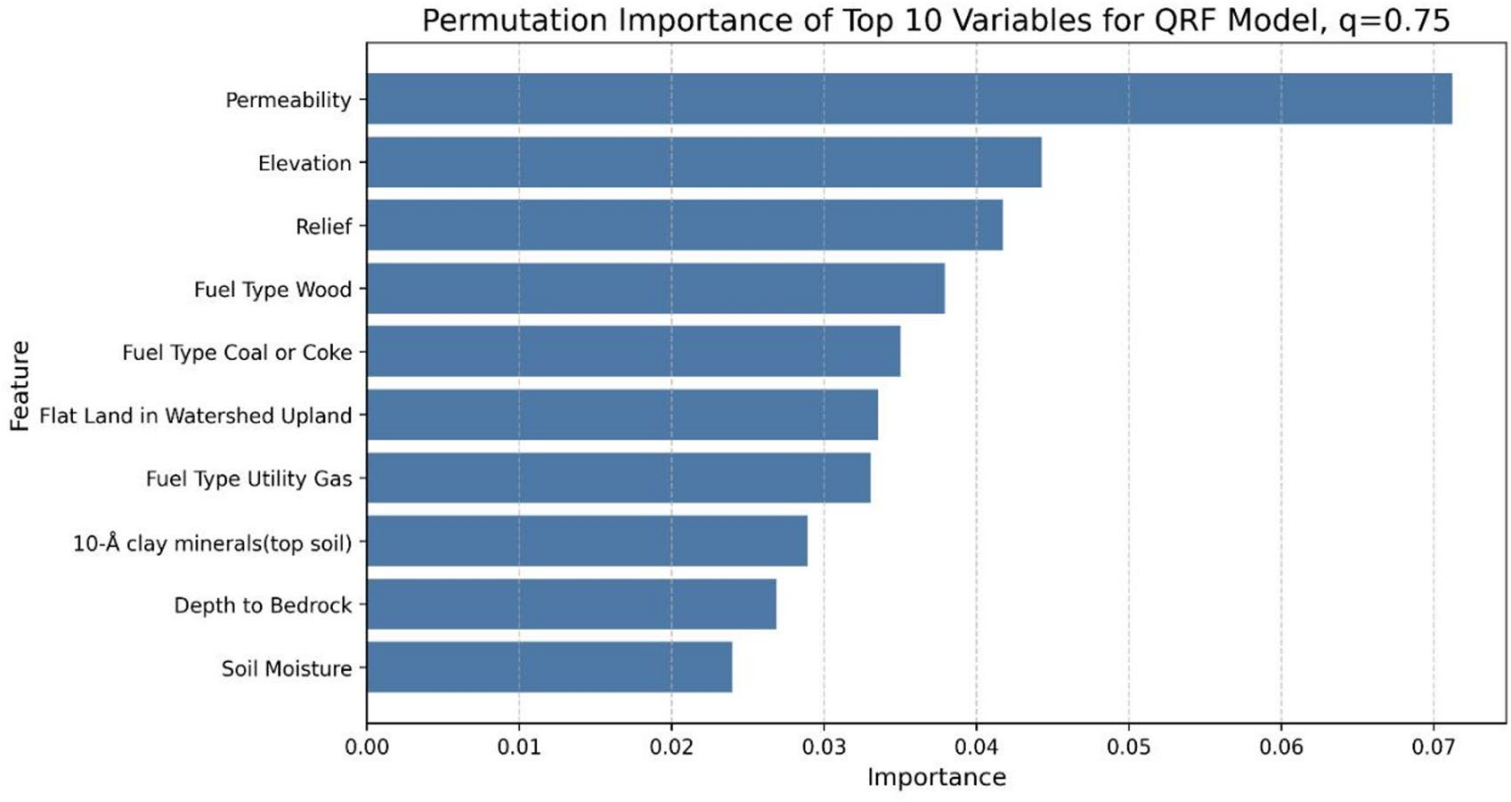
Permutation feature importance of top-10 variables for 75th percentiles of an individual QRF model.

**Fig. 13:**
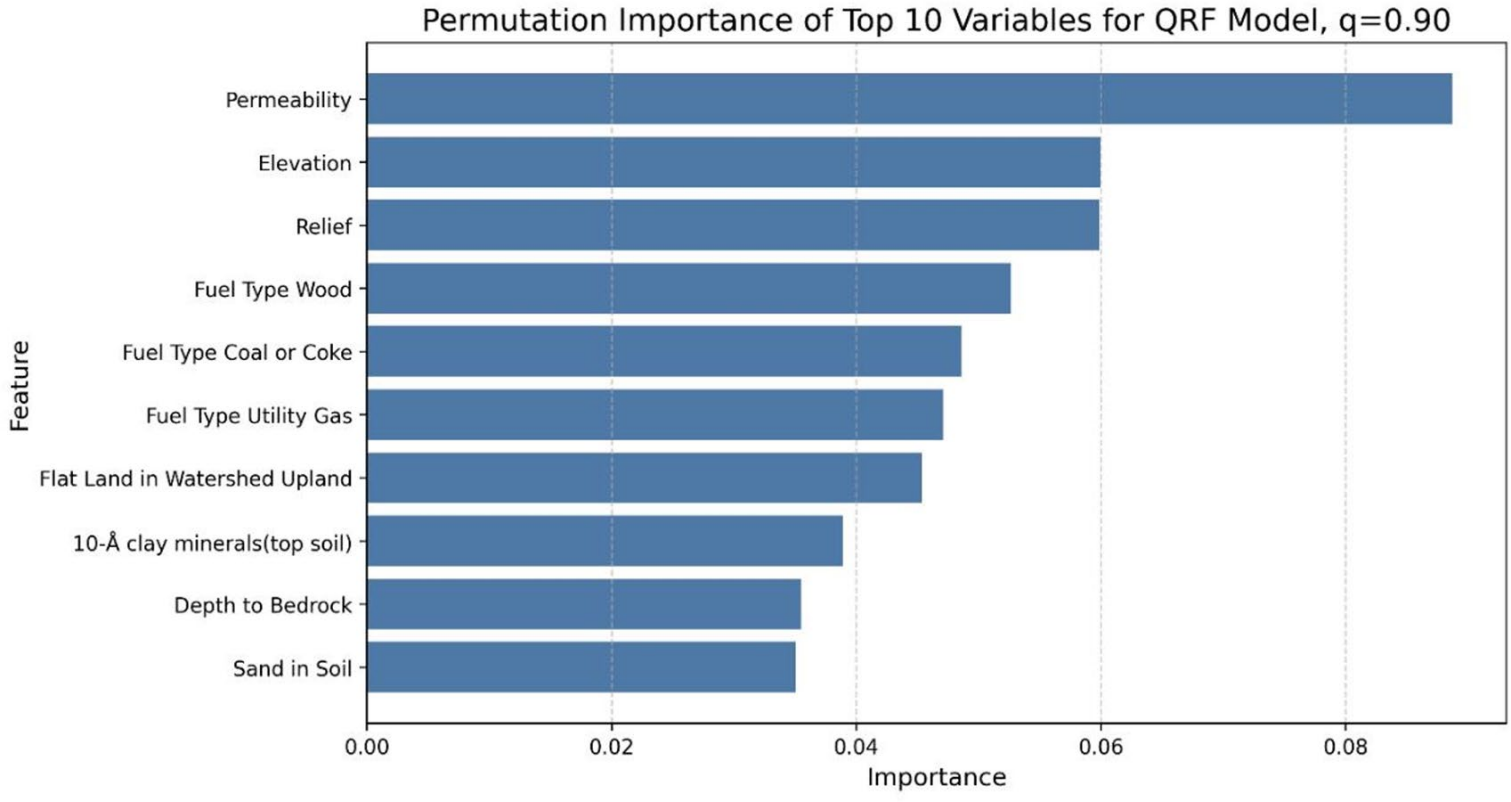
Permutation feature importance of top-10 variables for 90th percentiles of Individual QRF Model.

The importance of each variable on the QRF model was analyzed with permutation importance, and the permutation importance of the model was calculated for the 50th, 75th, and 90th percentiles (Figs. 11, 12 and 13, respectively). The analysis revealed notable trends, including a decrease in the importance of temperature as the target percentile increased. In contrast, the importance of permeability, elevation, and relief remained relatively consistent across different percentiles.

**Fig. 14:**
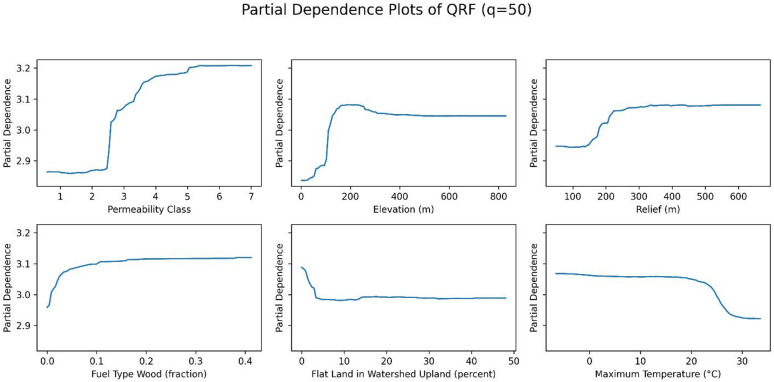
PDPs of six variables (permeability, elevation, fuel type wood, relief, depth to any soil restrictive layer, and the maximum temperature) at 50th percentile.

**Fig. 15:**
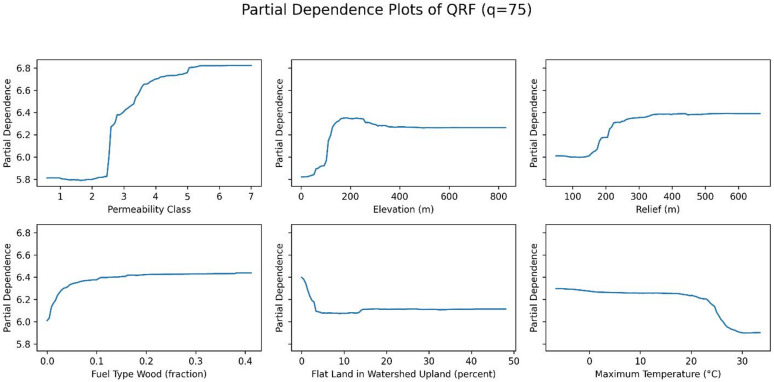
PDPs of six variables (permeability, elevation, fuel type wood, relief, depth to any soil restrictive layer, and the maximum temperature) at 75th percentile.

**Fig. 16:**
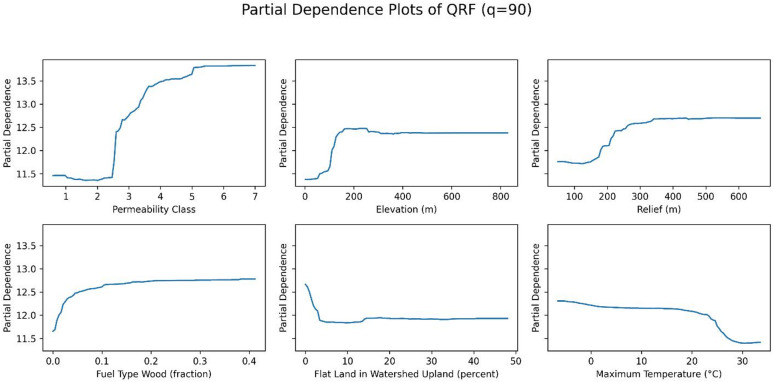
PDPs of six variables (permeability, elevation, fuel type wood, relief, depth to any soil restrictive layer, and the maximum temperature) at 90th percentile.

Figures 14, 15 and 16 show the partial dependence plots (PDPs) of six variables: permeability, elevation, relief, fuel type of wood, flat land in watershed upland, and maximum temperature, for the 50th, 75th, and 90th percentiles. The y-axis in each panel represents the change in the predicted radon concentration (pCi/L) as a function of a single predictor, holding all other predictors at their observed distribution. Note that the y-axis limits are chosen separately for each panel to make within-plot variation visible; therefore, absolute PDP amplitudes should not be compared directly across quantiles.

For permeability, elevation, and relief, the shape of the PDPs is relatively stable across the three quantiles, indicating that these variables have a similar directional effect on both median and upper-tail radon concentrations. For maximum temperature, permutation importance is highest at the 50th percentile and decreases at the 75th and 90th percentiles, even though the PDP for the 90th percentile spans slightly larger absolute changes on the y-axis. This reflects the fact that permutation importance quantifies the loss in out-of-sample predictive performance when a variable is permuted, and is therefore a relative measure of how much that variable contributes to explaining variation in a given quantile, rather than a direct function of the vertical range of the PDP. In other words, maximum temperature still influences higher quantiles, but its contribution to predictive accuracy becomes smaller compared with other predictors at the 75th and 90th percentiles. Together, these patterns suggest that while some predictors (e.g., permeability, elevation, and relief) act similarly across the distribution, others (e.g., maximum temperature) play a relatively larger or smaller role depending on whether median or extreme radon levels are being predicted.

The Individual QRF Model effectively identified communities with a high likelihood of elevated radon levels. This model provided the added ability to predict various quantiles of radon levels, which was notably useful for identifying areas at higher risk for extreme exposures. The Relative Variability Model was able to identify the areas that might have high variability but was unable to point out the specific pattern or distribution of the radon level in the community.

When revisiting the ZCTAs of high average and high variability analyzed with the Average Model and the Relative Variability Model, the 90th percentile to 50th percentile ratio of January predicted from the QRF model are 5.80 (A), 5.28 (B), and 5.64 (C). ZCTAs of high average and low variability showed 4.37 (D), 4.16 (E), and 4.78 (F) for the 90th percentile to 50th percentile ratio of January prediction. The ratio of high average and high variability ZCTAs tends to be higher than the ratio of high average and low variability ZCTAs. Furthermore, the QRF model can provide detailed information about what cannot be provided from the Average Model and the Relative Variability Model.

**Fig. 17:**
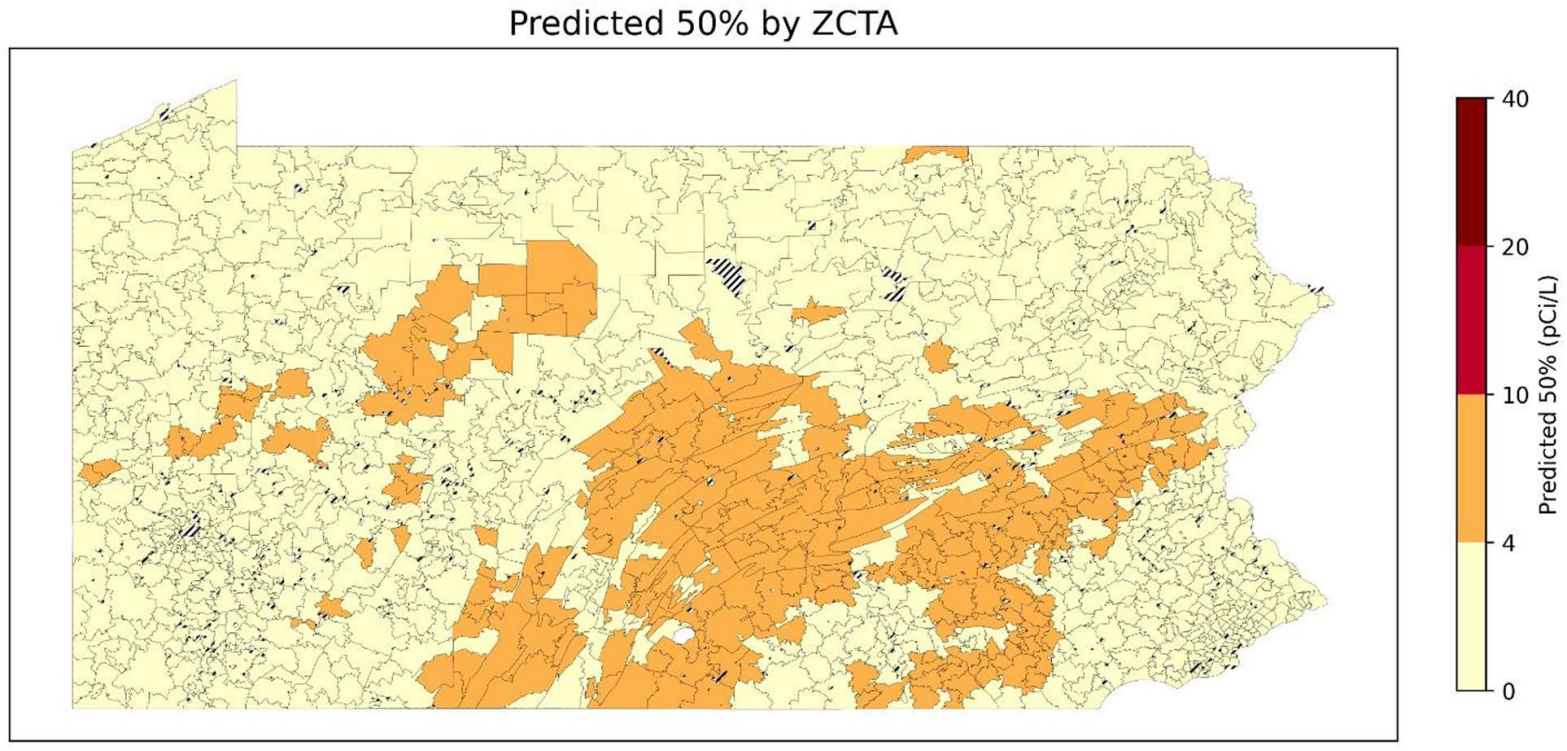
Predicted 50th percentile of radon level of January by ZCTA (ZCTAs where populated). Striped areas indicate ZCTAs where data is not available. Map was created in Python using GeoPandas^45^ and Matplotlib^46^ with ZCTA boundaries from the 2020 U.S. Census Bureau TIGER/Line shapefile^47^.

**Fig. 18:**
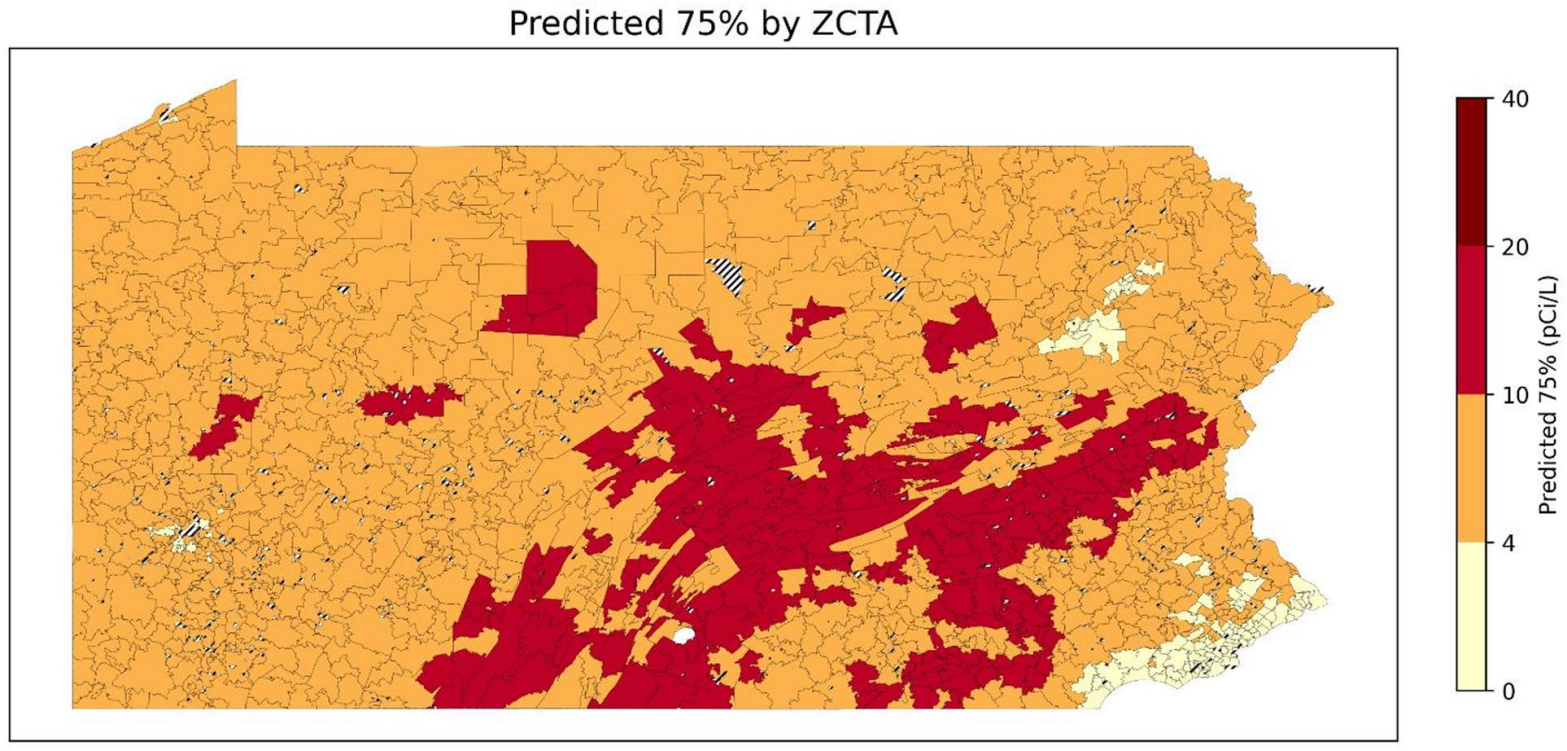
Predicted 75th percentile of radon levels for January by ZCTA (ZCTAs where populated). Striped areas indicate ZCTAs where data is not available. Map was created in Python using GeoPandas^45^ and Matplotlib^46^ with ZCTA boundaries from the 2020 U.S. Census Bureau TIGER/Line shapefile^47^.

Fig 17,18,19 show the predicted 50th, 75th, and 90th percentile radon levels by ZCTAs. When classifying predicted January radon levels into 0–4 pCi/L, 4–10 pCi/L, 10–20 pCi/L, and 20 + pCi/L, these results suggest that even areas with median levels of radon below 4 pCi/L can have a large percentage of residential radon test results with levels higher than 20 pCi/L. Comparing the overall level of the 50th percentile to 90th percentile, it shows that the radon levels can exhibit a high level of variability at relatively small geographic scales (e.g., ZCTAs). Furthermore, some areas have a lower median radon level but have a higher 90th percentile radon level. This aspect highlights the importance of testing radon levels even if the overall radon level of a neighborhood is low.

**Fig. 19:**
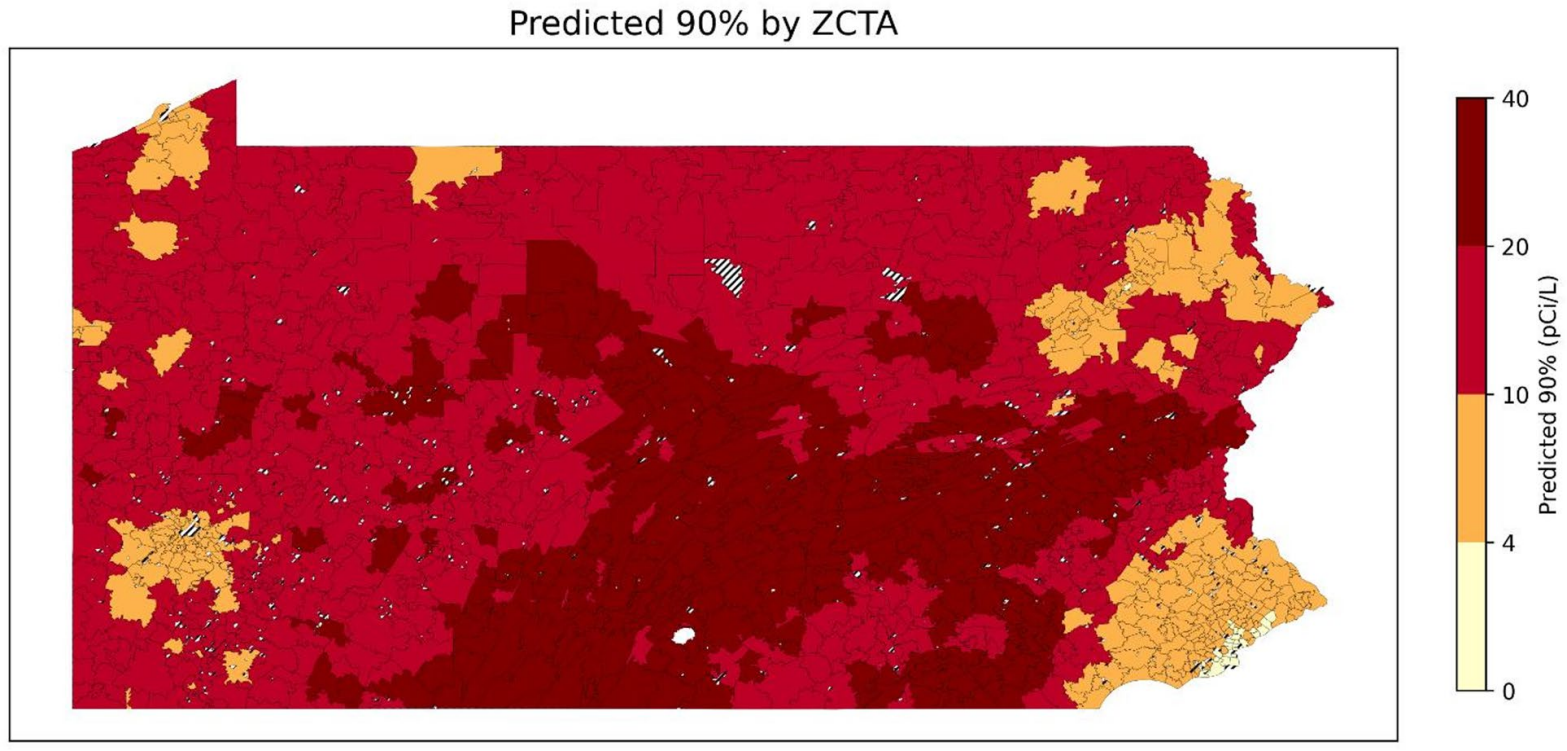
Predicted 90th percentile of radon levels for January by ZCTA (ZCTAs where populated). Striped areas indicate ZCTAs where data is not available. Map was created in Python using GeoPandas ^45^ and Matplotlib^46^ with ZCTA boundaries from the 2020 U.S. Census Bureau TIGER/Line shapefile^47^.

When comparing the different models, each offered distinct strengths in assessing radon exposure. For the Average Model, when evaluated against ZCTA–level mean radon concentrations, the model achieved RMSE values of approximately 2.7–3.2 pCi/L and explained about two-thirds of the between-ZCTA variability (R² = 0.67 for standard 5-fold CV and 0.53 for ZCTA-grouped CV). In contrast, when predictions were compared directly with individual house measurements, performance deteriorated substantially (R² = 0.10–0.12, MAPE > 160%), indicating that mean ZCTA estimates are not reliable proxies for the radon level in a given home. These results highlight that the Average Model is informative for population-level or ecological lung cancer risk assessments^68,69^, but insufficient for characterizing within-community heterogeneity or extreme exposures.

The Relative Variability Model directly addressed this limitation by modeling the coefficient of variation (CoV) of radon within a ZCTA. This model distinguished ZCTAs with relatively homogeneous radon levels (low CoV) from those with substantial within-area heterogeneity (high CoV), and identified environmental and soil-related drivers of that heterogeneity. In practical terms, ZCTAs with high predicted CoV correspond to communities where the mean radon level may appear moderate but a non-trivial fraction of homes can still experience very high indoor concentrations. These areas are natural priorities for intensified testing and targeted risk communication.

The Individual QRF Model further expanded the profiling of radon exposure by predicting multiple quantiles (50th, 75th, and 90th percentiles) of radon concentrations at the ZCTA level. Across these quantiles, the QRF achieved R² values from 0.54 to 0.67, with MAPE values generally between 19% and 30%. Thus, the model captured a substantial portion of the spatial variability in not only median exposure but also upper-tail radon levels. Upper percentile predictions (e.g., 75th and 90th percentile) identify ZCTAs where a sizeable fraction of homes are likely to exceed the EPA action level, providing a quantitative basis for prioritizing radon mitigation and outreach. Although percentage errors for the upper quantiles appear large when viewed in isolation, they are comparable to the inherent variability and measurement error in individual short-term radon tests. In practice, public health decision-making is driven less by the exact value of the 75th or 90th percentile and more by whether these upper-tail concentrations lie near or well above the EPA action level of 4 pCi/L. The QRF model is sufficiently accurate for this purpose, reliably distinguishing ZCTAs with modest elevations in radon from communities where a large share of homes are likely to experience substantially elevated concentrations, and is therefore best used as a tool for ranking and prioritizing areas for further testing and mitigation rather than as a substitute for household-level measurements.

By providing predictions at the ZCTA level, these models offered a more refined risk assessment tool compared to traditional county-level analyses. This granularity is necessary for identifying high-risk areas and overall risk of areas. Identifying and predicting variability or uncertainty in radon estimates within ZCTAs is crucial because radon levels can differ significantly even within the same community. These differences result in substantial uncertainties in estimated risks, highlighting the need for more tailored public health advisories and interventions that address the uncertainties in risk profiles not only across the communities but also within communities.

The Individual QRF Model’s detailed predictions are particularly useful for applications where extreme exposures are of concern. For example, ZCTAs with predicted 90th percentile radon levels well above 4 pCi/L can be prioritized for (i) more aggressive radon testing campaigns, (ii) subsidized mitigation programs, and (iii) tailored risk communication emphasizing the probability that a substantial share of homes exceeds the action level despite moderate mean values. In this way, the QRF output can be translated into concrete decision rules for allocating limited public health resources.

This study offers several strengths compared to previous studies. The use of multiple methods for characterizing ZCTA-level estimates of indoor radon exposure risk allows for more informed decision-making that can be directly applied to community-level interventions. By employing RF and QRF models, the analysis leveraged the capabilities of machine learning to handle complex datasets and nonlinear relationships. Multiple evaluation metrics, including RMSE, R², and MAPE, were utilized to ensure a comprehensive assessment of model performance. Notably, the Individual QRF Model offered unique capabilities that enabled a more thorough understanding of residential radon exposure and informed strategies for radiation protection and mitigation.

Although this study advances radon exposure modeling and demonstrates the utility of machine learning for community-level risk assessment, the models and data presented some limitations. The reliance on ACS and DEC data assumed uniform distribution of variables, such as housing characteristics and demographics, across ZCTAs. This assumption may oversimplify local variability and fail to reflect the nuanced factors that influence radon exposure. Additionally, secondary use of indoor radon test results is subject to selection bias, because households that choose to test for radon may differ systematically from those that do not. Future studies that incorporate point-level data and more granular geographic information could address these limitations and enhance the models’ accuracy and applicability.

Additionally, our meteorological covariates were limited to variables available from the Daymet product (temperature, precipitation, snow water equivalent, and vapor pressure). Although pressure-driven and wind-driven flows are known to influence radon entry by modifying soil-gas transport and indoor-outdoor pressure differences, we did not have access to spatially and temporally resolved atmospheric pressure or wind fields at the ZCTA level. The absence of these variables may have left some variation in radon concentrations unexplained and likely contributes to the residual error in our models.

Future research should aim to integrate more detailed geographic and temporal data to enhance the accuracy of radon predictions. This could include finer-scale soil and building data as well as finer-scale and long-term radon measurements to account for temporal variability.

While this study focused on Pennsylvania, the methods developed can be applied to other regions with similar or different radon risk concerns. Expanding the geographic scope will validate the model’s applicability and help develop a comprehensive radon risk map for broader areas.

Integrating radon predictions with other environmental hazards, such as air pollution and water quality, could provide a more detailed view of environmental health risks. This approach would help in designing multifaceted public health strategies that address multiple environmental factors simultaneously.

Furthermore, with more granular radon-level predictions, follow-up studies could offer more resources for linking radon exposure to lung cancer incidence at the ZCTA-level, which was not achievable from the previous study.

## Conclusion

The study demonstrates the value of a deep characterization of potential indoor radon exposure with multiple machine learning models, particularly RF and QRF. Analyses from the Average Model indicated that average radon concentrations by ZCTA (zip code tabulation area) were most strongly associated with subsurface permeability and heating fuel type. Because heating fuel mix is also closely related to the degree of urbanization and the underlying building type (e.g., greater use of wood heating in rural areas and greater reliance on utility-supplied gas in more urbanized ZCTAs), these associations should be interpreted with caution. In contrast, the Relative Variability Model showed that uncertainty in the estimated average indoor radon exposure level was greatest in ZCTAs with high within-area variability in elevation, saturated hydraulic conductivity, maximum temperature, and soil drainage. Individual QRF models further indicated that median and upper-quantile (75th and 90th percentile) radon concentrations tended to be higher in ZCTAs with high soil permeability, high elevation and relief (variation in vertical elevation in an area), high reliance on wood heating, and low proportion of flat land in watershed upland, thereby identifying areas where many households are likely to experience extreme radon exposure. Partial dependence plots also indicated that the influence of maximum temperature varied across quantiles, with stronger effects on median concentrations and attenuated effects at higher quantiles, potentially suggesting in areas with high radon potential, the temporal variability in exposure may be dampened. Overall, our findings demonstrate that in-depth analysis of both average exposure levels and their uncertainty at the ZCTA level can inform more effective testing, mitigation, and communication strategies. They also underscore the importance of updating radon estimates as new data are collected, expanding radon testing and data dissemination, and advancing modeling techniques to improve radon exposure estimation. Future research should further refine these models and extend their application to broader geographic regions and other environmental hazards to enhance environmental health risk assessment.

## Supplementary Information

Below is the link to the electronic supplementary material.

Supplementary Material 1

## Data Availability

Indoor radon measurement data for Pennsylvania were obtained from the Pennsylvania Department of Environmental Protection (PA DEP) which is publicly available.All datasets used in this study are publicly available from the original data providers: indoor radon measurement from the Pennsylvania Department of Environmental Protection, elevation from the USGS GMTED2010 product, soil characteristics from the USDA NRCS gNATSGO database, geochemical variables from the USGS Geochemical and Mineralogical Survey, hydrologic landscape data from USGS, meteorological variables from the Daymet database, and demographic and housing characteristics from the U.S. Census Bureau (Decennial Census and American Community Survey). Detailed information on data sources and preprocessing workflows is provided in the method paper. 44Data are available from the authors upon reasonable request.
