# Supplementary material for: Quantifying mean, variability, and uncertainty in indoor radon exposure in Pennsylvania using random forest and quantile regression forest models
Source: Sci Rep. 2026 Mar 5;16:15192. doi: 10.1038/s41598-026-37891-3 (PMC13179336; doi:10.1038/s41598-026-37891-3)
Supplement: Supplementary file 1 — Supplementary Material 1 [file 41598_2026_37891_MOESM1_ESM.docx]

Appendix A. Definitions of Model Evaluation Metrics

A.1. Root Mean Square Error (RMSE)

Let r_i_ denote the observed outcome and p_i_ refer the corresponding model prediction for observation i. The root mean square error (RMSE) is defined as

$$RMSE= \sqrt{\frac{1}{n}\sum_{i=1}^{n} \left( r_{i}-p_{i} \right)^{2}}$$

A.2. Coefficient of Determination (R²)

The coefficient of determination R² compares the residual sum of squares to the total sum of squares. It is defined as

$$R^{2}=1-\frac{\sum_{i=1}^{n} (r_{i}-p_{i})^{2}}{\sum_{i=1}^{n} (r_{i}-m)^{2}}$$

where m is the sample mean of the observed values r_i_. Higher values of R² indicate that a larger proportion of the variability in m is explained by the model.

A.3. Mean Absolute Percentage Error (MAPE)

The mean absolute percentage error (MAPE) quantifies the average relative prediction error as a percentage. For observations with r_i_ ≠ 0, it is defined as

$$MAPE=\frac{100}{n}\sum_{i=1}^{n} \left| \frac{{r_{i}-p}_{i}}{r_{i}} \right|$$

In practice, observations with r_i_ =0 are excluded from the MAPE calculation to avoid division by zero.

Appendix B. Individual Random Forest Model and QRF Model without variability variables

Another random forest (RF) model that uses the individual-level data was investigated to test the adoptability of predicting the radon level of individual houses by using the aggregated independent variables.

For this model, RF was used similarly to the previous Average Model and Relative Variability Model. Performance metrics such as root mean square error (RMSE), R-squared (R²), and mean absolute percentage error (MAPE) were used to evaluate the model's performance, and the evaluation was conducted by using 5 iterations of group 5-fold cross-validation (CV) with zip-code tabulation area (ZCTA) as the grouping variable. (Table A1) The metrics indicated poor predictive performance, suggesting significant variability in radon levels within each ZCTA, similar to the Average Model. Predicting high-variability measures from aggregated variables resulted in poor accuracy and highlighted the need for non-aggregated variables or other approaches.

Table B1. Metrics and their standard deviation of the individual RF model.

|  | **5-fold CV** | **Group 5-fold (ZCTA) CV** |
| --- | --- | --- |
| RMSE | 8.57 (0.11) | 8.60 (0.67) |
| R^2^ | 0.0054 (0.0010) | -0.0074 (0.0052) |
| MAPE | 95.66 (0.26) | 97.87 (2.49) |

Despite using individual-level data, this model had limitations on the prediction accuracy. This limitation was caused by the lack of point-level addresses for the residents and forced the use of ZCTA-level aggregated variables. Because the RF model inherently tended to predict average value, the benefit of incorporating individual-level data was diminished. Although poor performance metrics might imply the model is ineffective, they mainly highlight the challenges and limitations of modeling radon exposure with insufficient spatial details. The results show that the prediction of highly variable radon levels cannot be achieved from the aggregated variables. To fully utilize the information from the dataset, other approaches should be considered (e.g., QRF).

We also evaluated an alternative QRF model that included only ZCTA-level mean covariates (i.e., without any variability/entropy predictors), mirroring the “average-only” specification commonly used in previous radon mapping studies. The model was trained and validated using the same procedures as the main QRF model described in the text. As summarized in Table B2, predictive performance of the mean-only QRF was very similar to that of the full model with variability covariates, with nearly identical RMSE, R², and MAPE values across the 50th, 75th, and 90th percentiles. This suggests that, while variability measures are informative for understanding within-ZCTA heterogeneity and for the Relative Variability Model, they contribute relatively little additional signal for predicting ZCTA-level radon quantiles beyond what is already captured by the mean-level covariates.

Table B2. Metrics and standard deviation of individual quantile regression forest (QRF) model without variability data.

|  | **50th** | | **75th** | | **90th** | |
| --- | --- | --- | --- | --- | --- | --- |
|  | 5-fold CV | Group 5-fold (ZCTA) CV | 5-fold CV | Group 5-fold (ZCTA) CV | 5-fold CV | Group 5-fold (ZCTA) CV |
| **RMSE** | 1.71 (0.026) | 1.98 (0.32) | 3.76 (0.046) | 4.44 (0.51) | 7.14 (0.064) | 8.39 (0.58) |
| **R^2^** | 0.51 (0.013) | 0.35 (0.024) | 0.61 (0.0078) | 0.46 (0.05) | 0.66 (0.0059) | 0.52 (0.032) |
| **MAPE** | 19.57 (0.15) | 21.37 (1.37) | 23.21 (0.22) | 30.19 (1.48) | 31.70 (0.62) | 42.31 (1.90) |

Appendix C. Sensitivity of model performance by sample size, season, and region

To evaluate how prediction performance varied with data density and spatiotemporal context, we computed mean absolute percentage error (MAPE) for each model after stratifying ZCTA–month observations by (i) the number of radon tests, (ii) season, and (iii) metropolitan region (Philadelphia, Pittsburgh, other ZCTAs). Figures C1–C15 summarize these diagnostic analyses.

C.1. Average Model

For the Average Model, MAPE decreased steadily as the number of radon tests per ZCTA–month increased (Figure C1). Cells with 10–19 tests had a MAPE of about 31%, which improved to 24% for 20–29 tests and 20% for 30–49 tests. For ZCTA–months with ≥100 tests, MAPE stabilized around 13–14%, and was 13.0% in ZCTA–months with ≥200 tests. This pattern confirms that ZCTA-level average predictions are more reliable when based on a larger number of underlying measurements.


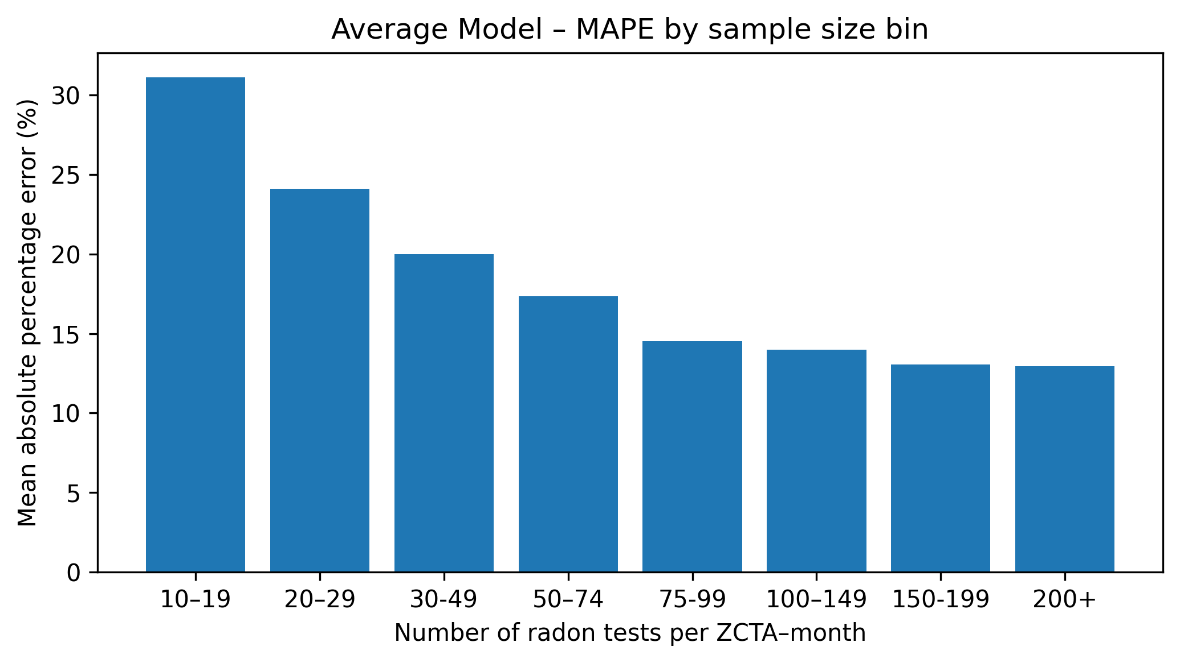


Figure C1. Mean absolute percentage error (MAPE) of the Average Model by sample size bin (number of radon tests per ZCTA–month)

When stratified by season, MAPE was lowest in spring (19.5%) and fall (18.2%) and higher in summer (23.2%) and winter (21.8%). (Figure C2)


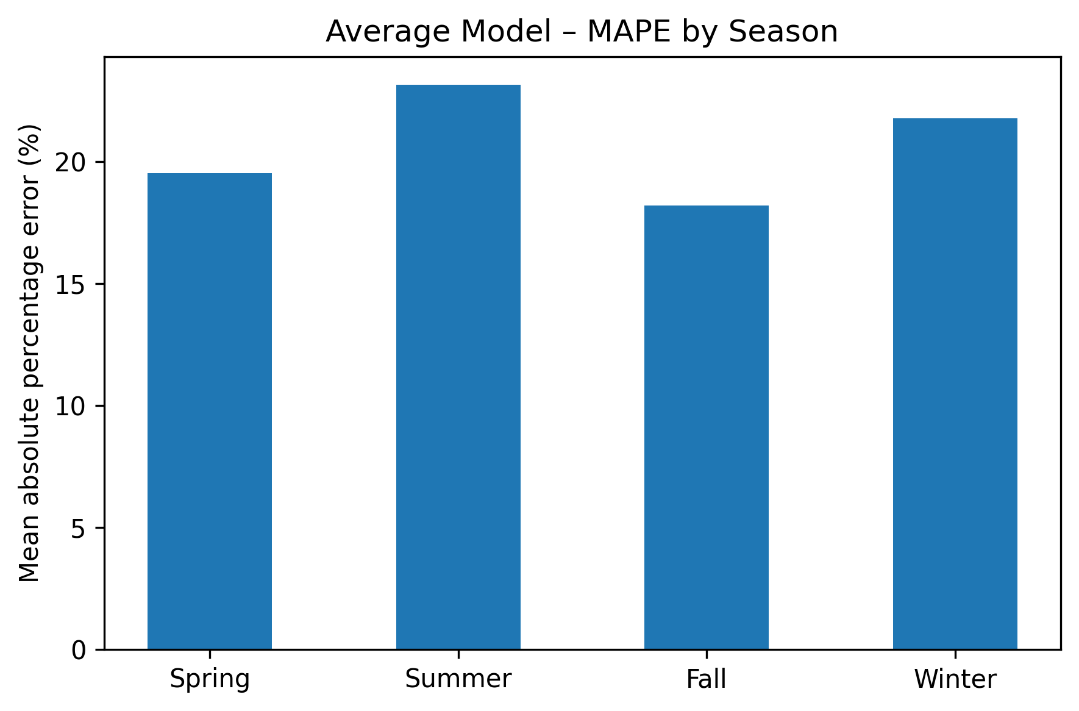


Figure C2. Mean absolute percentage error (MAPE) of the Average Model by season.

By metropolitan status, MAPE was lowest in Philadelphia ZCTAs (14.4%), intermediate in Pittsburgh (19.4%), and highest in other ZCTAs (24.0%). (Figure C3) This gradient largely mirrors the underlying distribution of sample sizes, with major metropolitan areas having more radon tests per ZCTA–month. The numbers of measures per ZCTA were 1409.3 (Philadelphia), 847.6(Pittsburgh), and 532.5 (Other), respectively. Thus, the apparent regional differences in accuracy likely reflect data density rather than systematic spatial bias in the Average Model.


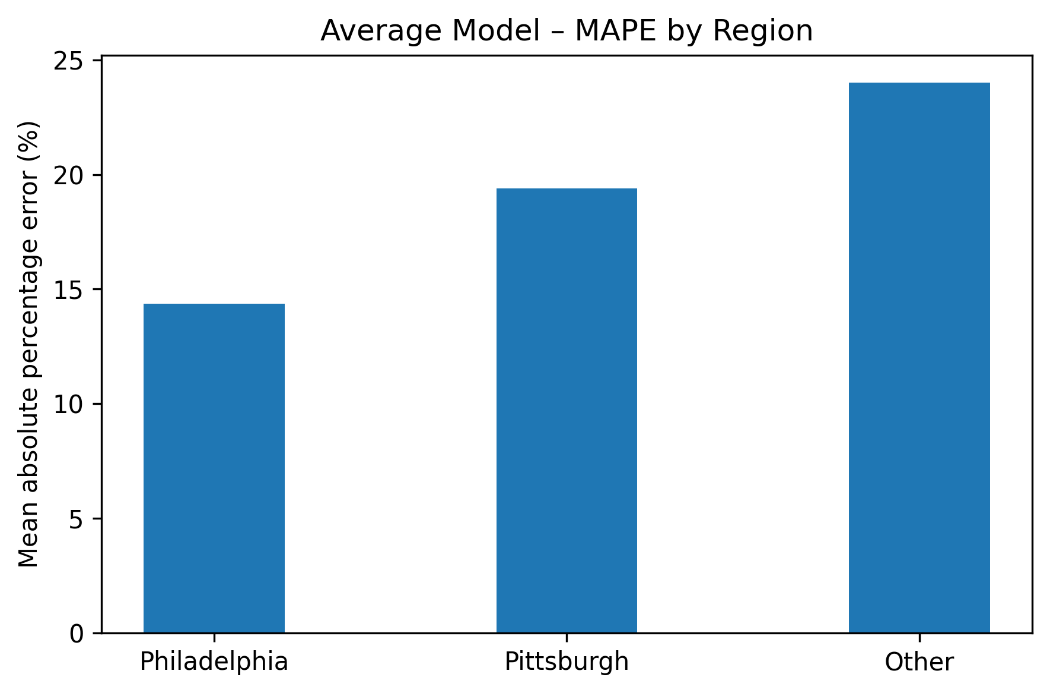


Figure C3. Mean absolute percentage error (MAPE) of the Average Model by metropolitan region (Philadelphia, Pittsburgh, other ZCTAs).

C.2. Relative Variability Model

For the Relative Variability Model, which predicts the coefficient of variation (CoV) of radon within each ZCTA–month, MAPE also decreased with increasing sample size. ZCTA–months with 10–19 tests had a MAPE of 27.4%, which improved to 19.9% for 20–29 tests and 17.2% for 30–49 tests. For ZCTA–months with ≥100 tests, MAPE dropped to 11–12%, and reached 9.0% when ≥200 tests were available. (Figure C4)


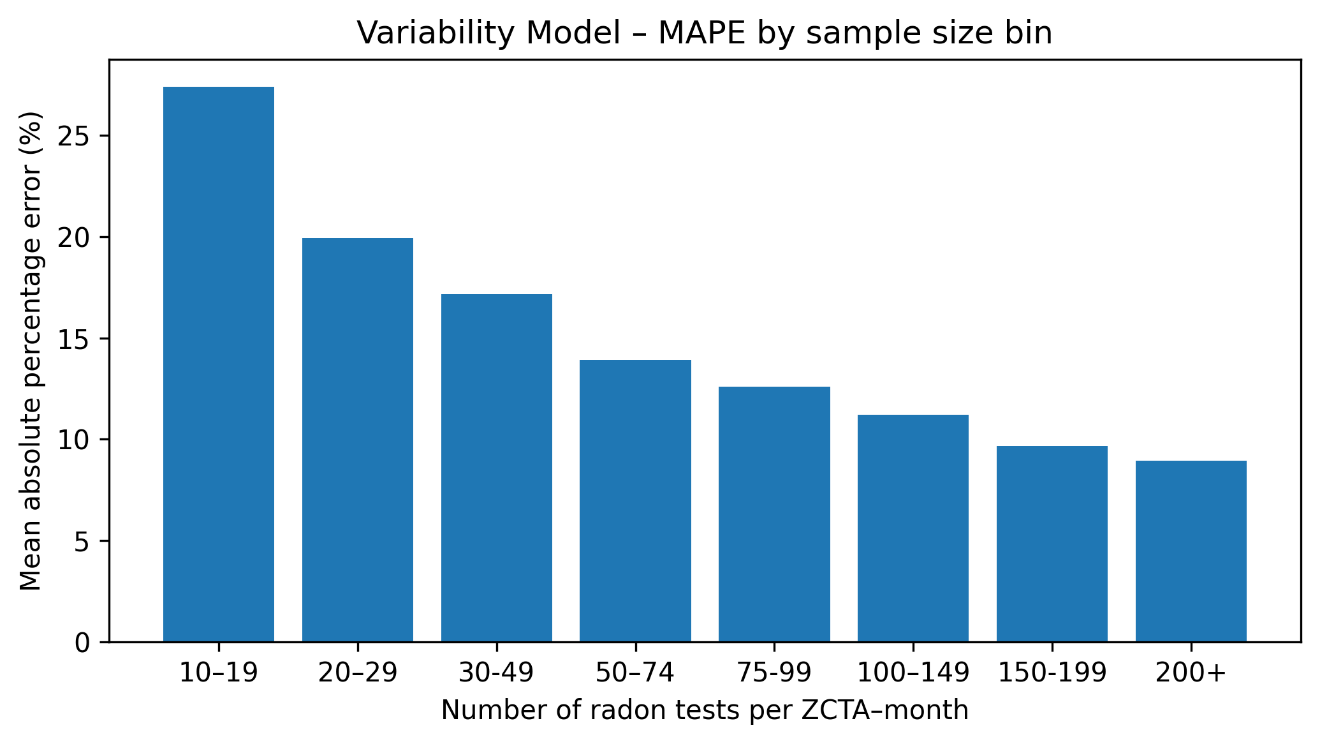


Figure C4. Mean absolute percentage error (MAPE) of the Relative Variability Model by sample size bin (number of radon tests per ZCTA–month).

Seasonal differences in MAPE were relatively modest for this model, ranging from 15.5% in spring to 17.4% in summer, with fall and winter around 16.8–17.0%. (Figure C5)


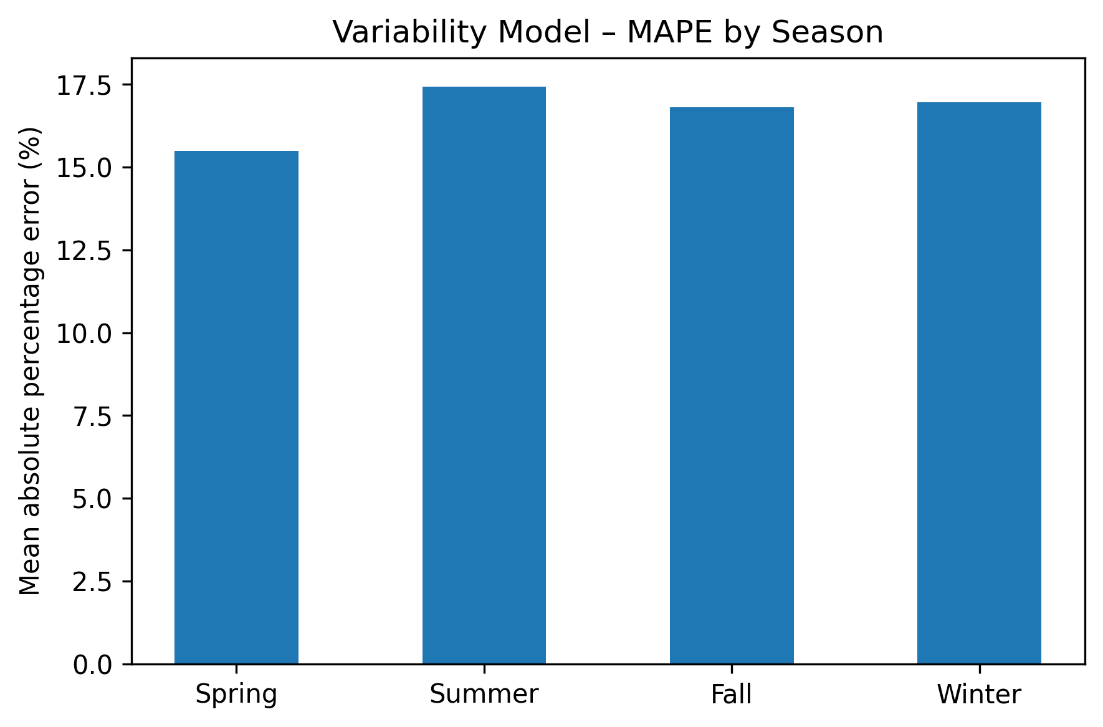


Figure C5. Mean absolute percentage error (MAPE) of the Relative Variability Model by season.

Region-specific MAPEs were 14.2% for Philadelphia, 16.4% for Pittsburgh, and 16.8% for other ZCTAs (Figure C6).


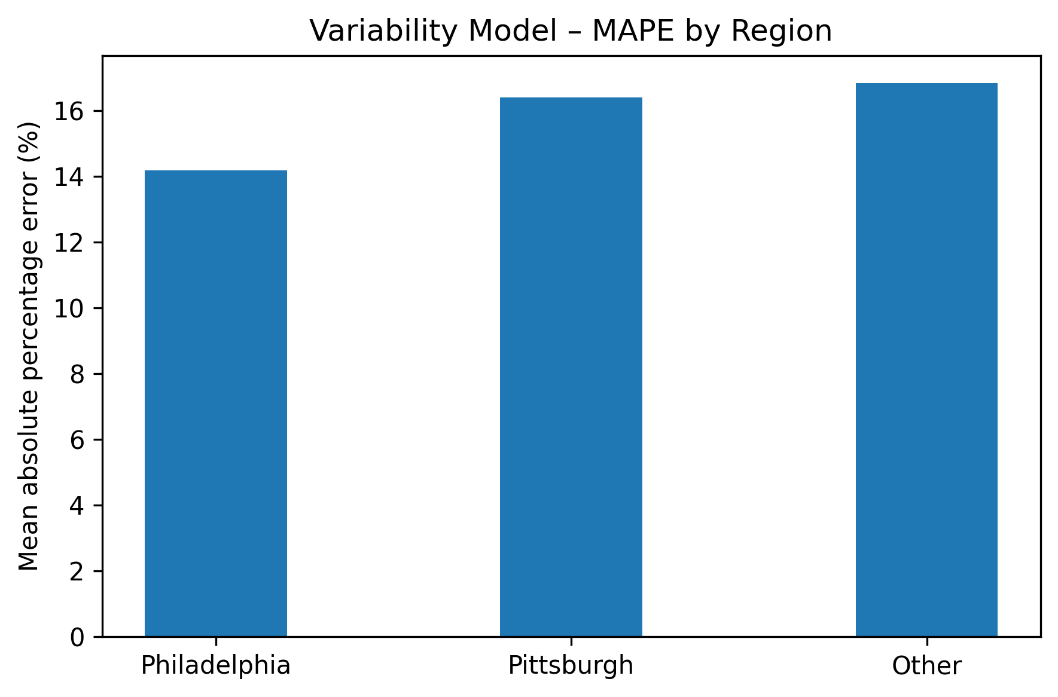


Figure C6. Mean absolute percentage error (MAPE) of the Relative Variability Model by metropolitan region (Philadelphia, Pittsburgh, other ZCTAs).

C.3. Individual QRF Models (50th, 75th, and 90th percentiles)

For all three QRF quantiles, MAPE decreased consistently as the number of tests per ZCTA–month increased. (Figure C7-C9)

– At the 50th percentile, MAPE was 31.2% for 10–19 tests, 22.8% for 20–29 tests, 20.0% for 30–49 tests, and declined to 11.4–12.3% for ZCTA–months with ≥100–200 tests.

– At the 75th percentile, the same pattern was observed but at a higher overall error level: 36.6% (10–19 tests), 28.2% (20–29), 22.6% (30–49), and 12.5–14.4% for ≥100 tests.

– At the 90th percentile, MAPE was highest, reflecting the greater difficulty of predicting extreme values: 50.4% for 10–19 tests, 39.1% for 20–29 tests, 30.6% for 30–49 tests, and 16.7–19.8% for ≥100–200 tests.

These results indicate that upper-tail predictions are inherently noisier, but that, in well-sampled ZCTA–months, even 75th–90th percentile estimates achieve MAPE values on the order of 15–20%.
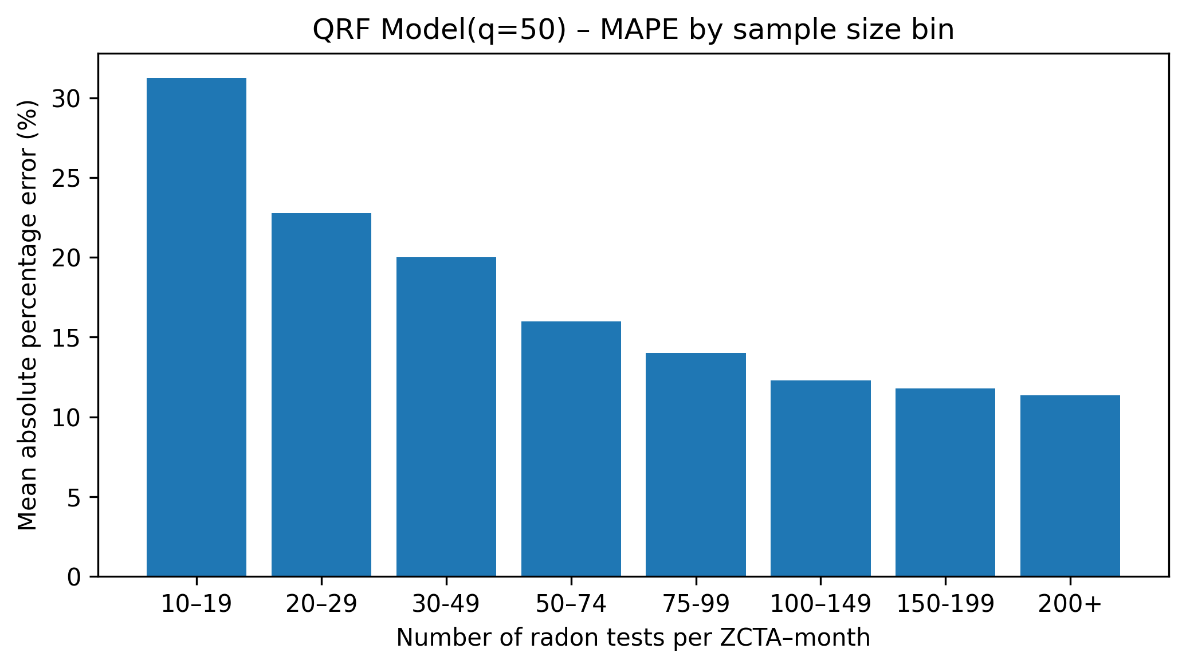


Figure C7. Mean absolute percentage error (MAPE) of the QRF Model for the 50th percentile by sample size bin (number of radon tests per ZCTA–month).


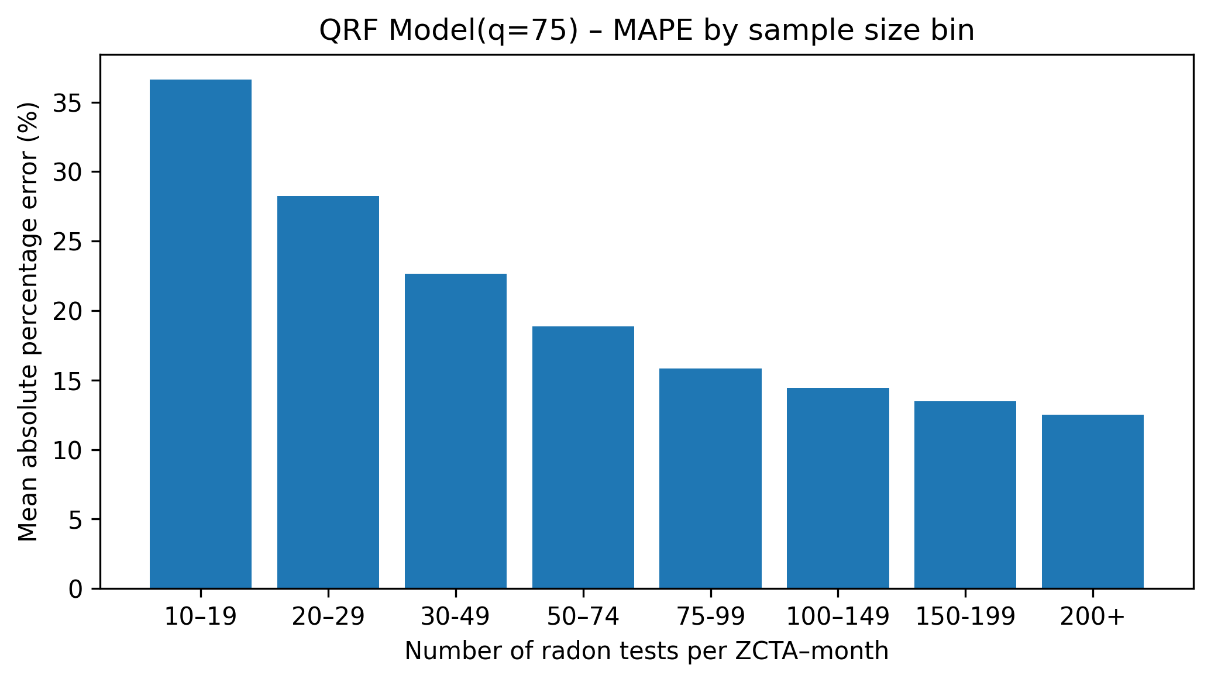


Figure C8. Mean absolute percentage error (MAPE) of the QRF Model for the 75th percentile by sample size bin (number of radon tests per ZCTA–month


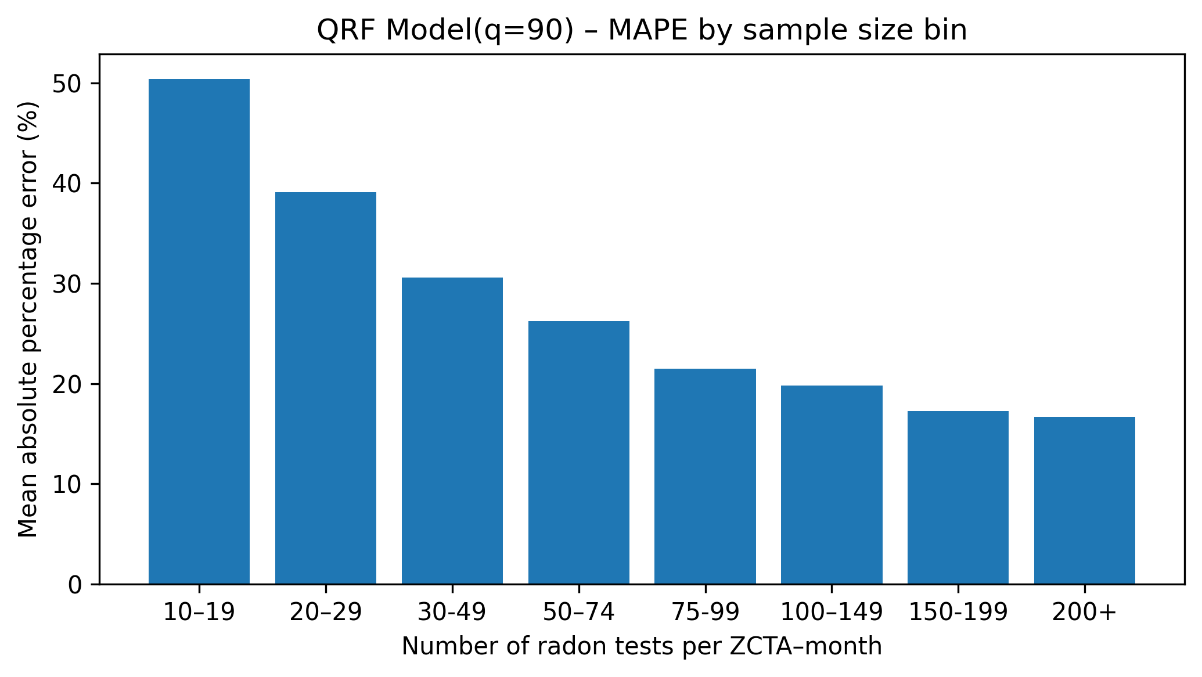


Figure C9. Mean absolute percentage error (MAPE) of the QRF Model for the 90th percentile by sample size bin (number of radon tests per ZCTA–month).

Seasonal stratification again showed higher MAPE in summer and winter than in spring and Fall. (Figure C10-C12) For the 50th percentile QRF, MAPE was 18–19% across seasons, with slightly higher errors in summer and winter. At the 75th and 90th percentiles, MAPE increased overall (e.g., for q=90: 25.9% in fall vs 30–33% in other seasons), but the pattern of better performance in the shoulder seasons and larger errors during summer and winter persisted. This is consistent with stronger seasonal variability and possibly more heterogeneous behaviors during the heating and cooling seasons.
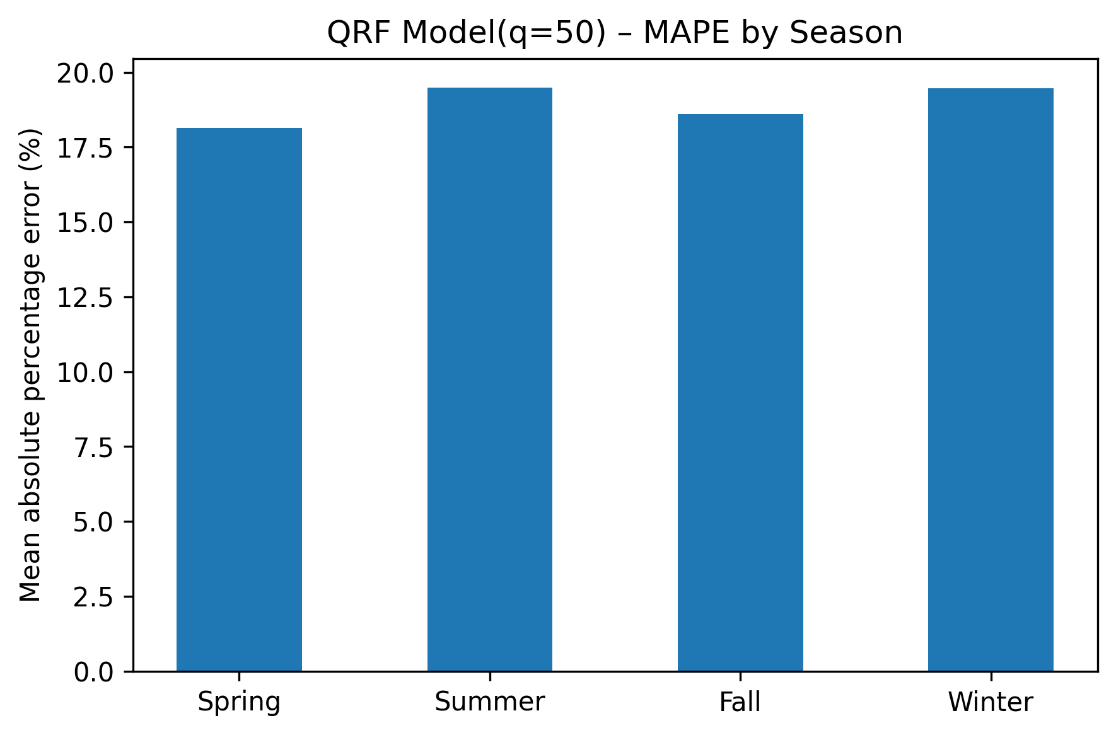


Figure C10. Mean absolute percentage error (MAPE) of the QRF Model for the 50th percentile by season.


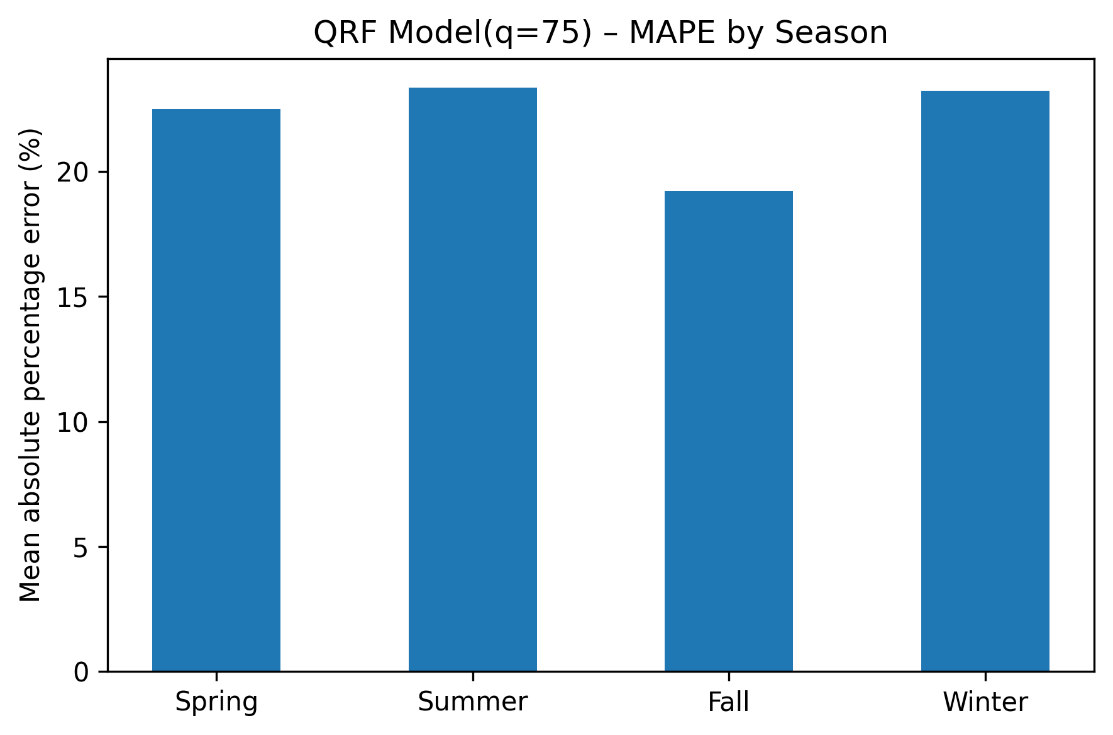


Figure C11. Mean absolute percentage error (MAPE) of the QRF Model for the 75th percentile by season.


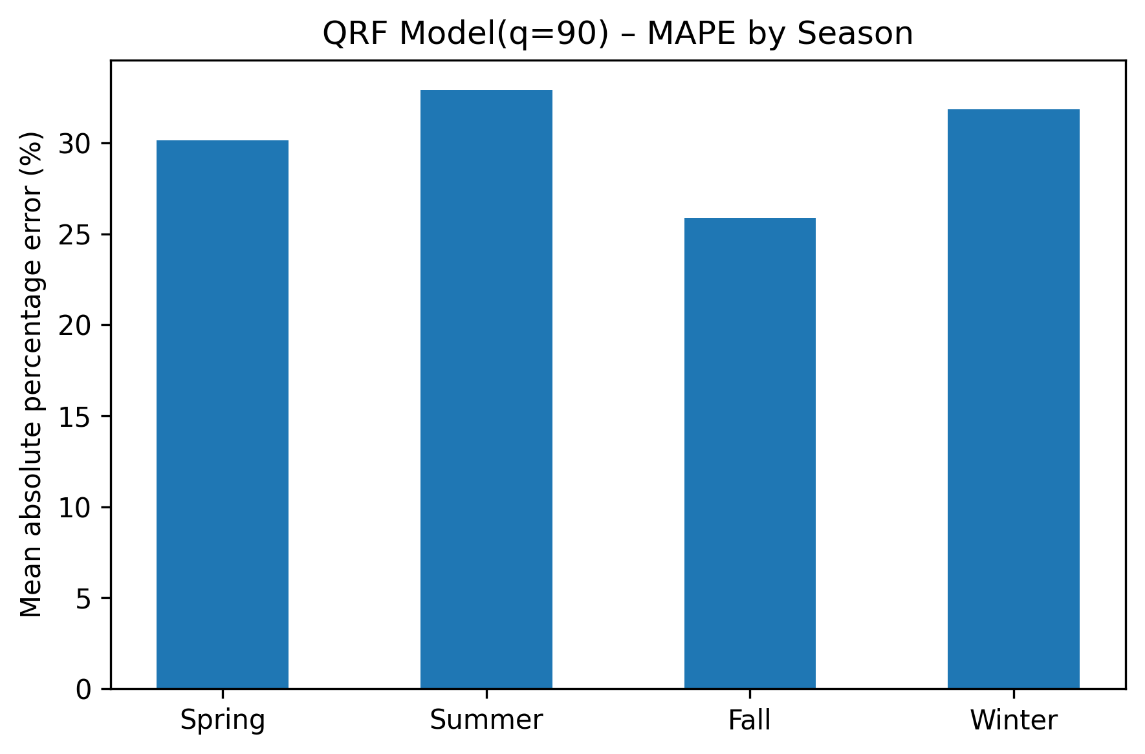


Figure C12. Mean absolute percentage error (MAPE) of the QRF Model for the 90th percentile by season.

Across quantiles, metropolitan areas again tended to show smaller MAPE than other ZCTAs (Figure C13-C15). For the 50th percentile QRF, MAPE was 12.6% in Philadelphia, 15.7% in Pittsburgh, and 22.8% in other ZCTAs. For the 75th percentile, the corresponding values were 14.9%, 21.0%, and 25.6%, and for the 90th percentile, 20.7%, 32.0%, and 33.8%. As with the Average Model, this pattern aligns with differences in sample size: Philadelphia and Pittsburgh ZCTAs have more radon tests, leading to more stable quantile estimates. The QRF performance therefore, appears primarily constrained by data density rather than by intrinsic regional bias.


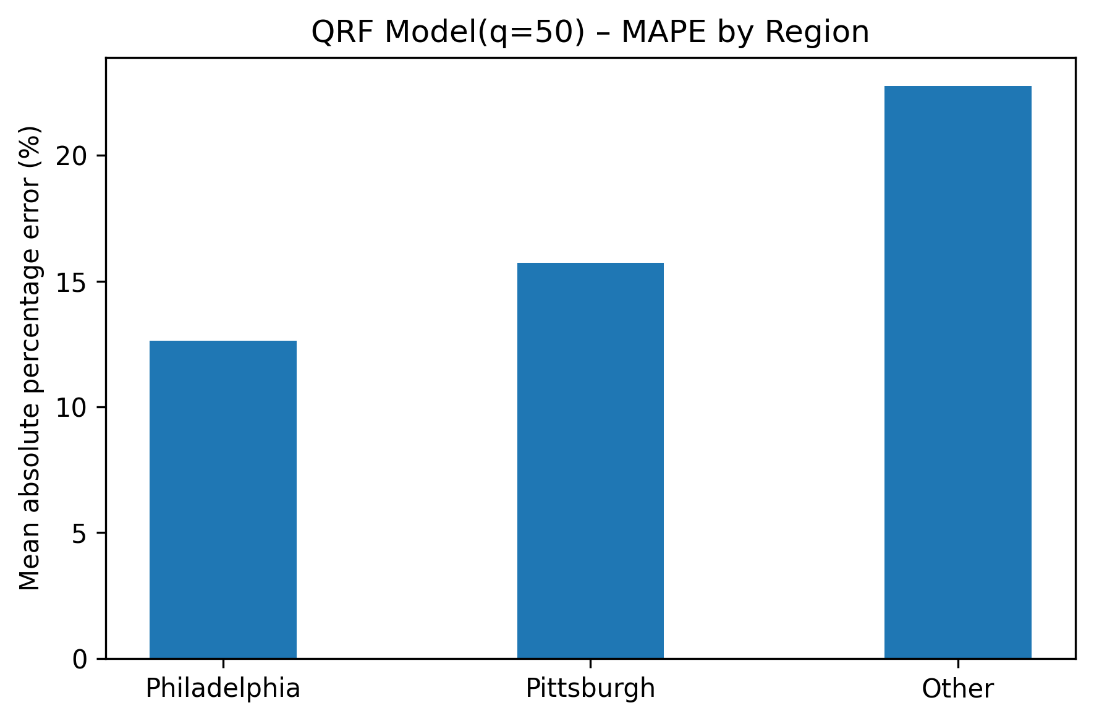


Figure C13. Mean absolute percentage error (MAPE) of the QRF Model for the 50th percentile by metropolitan region (Philadelphia, Pittsburgh, other ZCTAs).


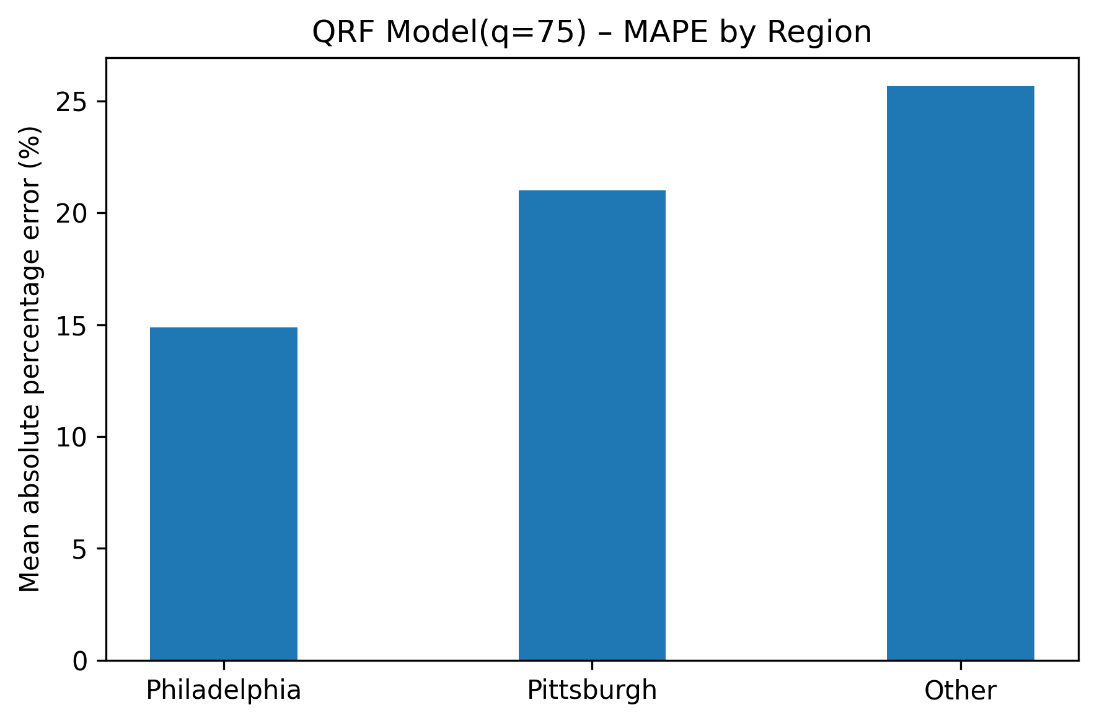


Figure C14. Mean absolute percentage error (MAPE) of the QRF Model for the 75th percentile by metropolitan region (Philadelphia, Pittsburgh, other ZCTAs).


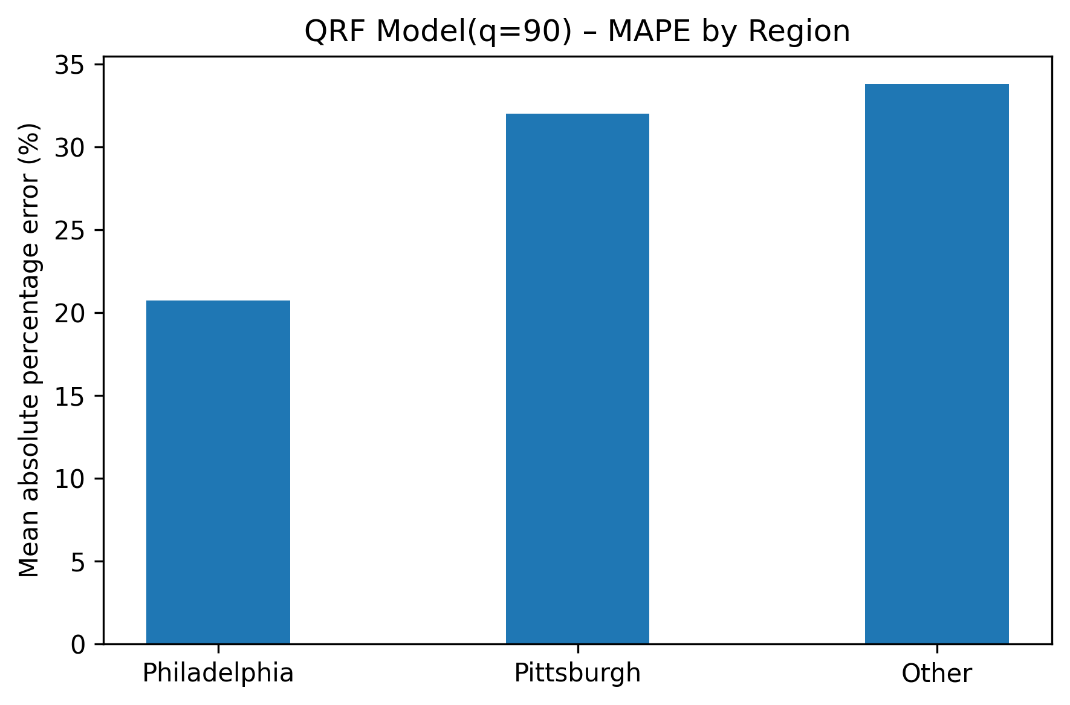


Figure C15. Mean absolute percentage error (MAPE) of the QRF Model for the 90th percentile by metropolitan region (Philadelphia, Pittsburgh, other ZCTAs).
